# Transcriptome analysis reveals effects of ethynylestradiol and bisphenol A on multiple endocrine and metabolic pathways in the pituitary and liver of female Atlantic cod (*Gadus morhua*)

**DOI:** 10.3389/fendo.2024.1491432

**Published:** 2025-01-27

**Authors:** Fekadu Yadetie, Xiaokang Zhang, Anna Reboa, Gwenaëlle Samantha Chloe Noally, Mariann Eilertsen, Mitchell Stewart Fleming, Jon Vidar Helvik, Inge Jonassen, Anders Goksøyr, Odd André Karlsen

**Affiliations:** ^1^ Department of Biological Sciences, University of Bergen, Bergen, Norway; ^2^ Department of Informatics, University of Bergen, Bergen, Norway

**Keywords:** endocrine disruptors, energy homeostasis, fish reproduction, lipid homeostasis, gonadotropins

## Abstract

**Introduction:**

The pituitary and liver are among the main sites of action of estrogens in fish. Years of research has shown that xenoestrogens can interfere with functions of estrogens. There is however incomplete understanding of xenoestrogen targets genes, their molecular mechanisms and potential effects in some of the target organs, particularly the pituitary.

**Methods:**

We performed a comprehensive analysis of pituitary and liver transcriptome 72 h after injection of ethynylestradiol (EE2: 10, 50 or 250 nmol/kg body weight/bw) and bisphenol A (BPA: 8, 40 or 200 μmol/kg bw) in juvenile female Atlantic cod (*Gadus morhua*).

**Results:**

A broad range of reproductive and metabolic pathways were affected in both organs by BPA and EE2. In the pituitary, effects on the expression of many genes associated with reproduction-related hormonal pathways including the gonadotropin system, as well as genes in processes such as cell differentiation and metabolic homeostasis were observed. In the liver, in addition to upregulation of well-known estrogen marker genes, effects on metabolic pathways, in particular, a coordinated downregulation of genes in the triglyceride synthesis pathways were observed.

**Discussion:**

The results suggest that estrogenic compounds affect a broad range of reproductive and metabolic processes in the pituitary. The alterations in the liver unravel the transcriptional changes underlying metabolic remodeling during estrogen induced vitellogenesis. This study provides new insights into mechanisms of endocrine and metabolic interactions that can be potential targets of environmental estrogens in fish. The study also identifies potential gene expression biomarkers for pituitary and liver effects of xenoestrogens.

## 1 Introduction

Estrogens regulate many reproductive processes in fish and other vertebrates. In fish, the brain, pituitary and liver are among the main sites of action of estrogens in the brain-pituitary-gonadal-hepatic axis. Under the influence of brain factors, the pituitary synthesizes gonadotropins, which, in the female fish, stimulate synthesis of estradiol that in turn stimulates vitellogenesis in the liver (1, 2). Estrogens also modulate gonadotropin synthesis and release through feedback mechanisms in the brain and pituitary (3–5). Therefore, estrogenic compounds acting on the estrogen target tissues such as the liver and the pituitary have the potential to interfere with the reproductive processes in fish.

In the last few decades, endocrine disrupting chemicals (EDCs) in the environment, especially estrogen mimicking anthropogenic compounds (xenoestrogens), have caused concerns for their potential effects on human and wildlife health (6, 7). Industrial compounds such as the plasticizer bisphenol A (BPA) and the pharmaceutical estrogen ethynylestradiol (EE2) are among widely studied endocrine disruptors that are ubiquitous in the environment (8–12). BPA is a high production volume industrial chemical, used mainly in manufacturing of plastic products and epoxy resins used in, for example, surface coatings of food packaging metal cans (13). BPA has been shown to have estrogenic effects and may also cause metabolic disruption (14, 15). Recent studies also reported that many BPA alternatives have similar endocrine disrupting effects as BPA (13, 16). EE2 is used in contraceptive pills and often detected in domestic sewage and can contaminate the aquatic environment (17–19). EE2 is a potent estrogen, and numerous studies have documented its endocrine disruption effects such as increased synthesis of vitellogenin, feminization of male fish, reduced fertility and population decline (12, 20–26).

Most studies have investigated molecular effects of these EDCs in fish, largely using a limited set of biomarkers such as vitellogenin production (27, 28). While estrogen responsive biomarkers are informative on exposure to estrogens, they provide limited information on broader potential targets and processes affected. A few recent transcriptome-based studies have shown the potential of omics to identify new targets that may provide more insights into mode of action of EDC in the liver (29, 30). Studies on estrogen and xenoestrogen targets organs such as the pituitary are sparce (23), particularly using omics approaches. Therefore, mapping transcriptome responses to these compounds in the pituitary may reveal potential new targets and offer more mechanistic insights. Likewise, further transcriptome studies in the liver can offer insights into diverse pathways such as energy metabolism that may be affected by estrogens. Understanding potential effects on energy pathways can be important since the reproductive process is associated with major events of energy mobilization from the liver to the ovary (31, 32). Under estrogen stimulation, nutrients, mainly lipids, are transported from the liver to the ovary with vitellogenin to sustain embryos post fertilization (1, 2, 33). The liver is an important energy storage organ in many fish such as the Atlantic cod, where the liver constitutes of 50-60% lipids (34). Estrogens appear to play an important role in transcriptional processes during energy mobilization (29). In fact, estrogen stimulated increases in plasma lipid levels have been reported (35–37). However, little is known about the underlying transcriptome alterations during lipid mobilization in the liver, and further transcriptome studies will increase our overall understanding of mechanisms involved and potential endocrine perturbations by xenoestrogens. Omics approaches can generate data on genome-wide targets that enable assessment of effects on multiple cellular processes. Omics data can also indicate early molecular changes and biomarkers that can be predictors of apical adverse toxicological endpoints facilitating development of adverse outcomes pathways (23, 38–40). The primary aim of the current study is to investigate the effects of EE2 and BPA on global gene expression in both the pituitary and liver. The secondary aim is to identify new potential gene expression biomarkers for pituitary and liver effects in juvenile female *G. morhua*. Atlantic cod is one of the most important species in commercial fisheries in the North Atlantic Ocean, and there is an increasing interest for its aquaculture development (41, 42). It is also an important species in laboratory toxicological studies and in environmental monitoring (43–48). Its genome has been sequenced and annotated (49), which facilitates its use in omics-based studies. The Atlantic cod in coastal areas of Northen Europe can be exposed to environmental contaminants including xenoestrogens (50).

## 2 Materials and methods

### 2.1 Chemicals

DMSO (CAS No: 67-68-5), 17α-ethynylestradiol (CAS No: 57-63-6), bisphenol A (CAS No: 80-05-7), Kolliphor^®^ EL (CAS No: 61791-12-6) and all other chemicals, unless specified otherwise, were purchased from Sigma-Aldrich (Sigma-Aldrich, Oslo, Norway).

### 2.2 Fish husbandry

Atlantic cod (*G. morhua*) were obtained from Havbruksstasjonen in Tromsø AS (Nofima, Tromsø, Norway) and kept in 500 L tanks in 10°C, 34 ppt seawater with a 12 h light/l2 h dark cycle at the Industrial and Aquatic Laboratory (Bergen, Norway). Juvenile Atlantic cod of approximately 1.5 years old, body weight (bw) 332 ± 69g (mean ± standard deviation) were used for the experiment. The fish were fed with a commercial diet (Amber Neptune, batch no. 3343368, Skretting, Stavanger, Norway) until the start of the treatment. The fish were sexually immature, as confirmed during dissection (gonado-somatic index: 0.59 ± 0.12, mean ± sd). The experiment was performed in accordance with the guidelines and with approval of the Norwegian Board of Biological Experiments with Living Animals (FOTS ID 11730).

### 2.3 Exposure design

The fish were divided into 500 L tanks (16 fish per group) and acclimatized for one week and fed with 1% of their biomass daily until two days before the intraperitoneal (ip) injection. The fish were not fed during exposure. The fish were anesthetized using methacaine in sea water and weighed before injection. The exposure groups consisted of vehicle control, low, medium and high doses of each of EE2 and BPA. The compounds were dissolved in DMSO and mixed with the carrier (25% Kolliphor in PBS) to prepare the highest concentrations from which the medium and low concentrations were prepared by dilution in the carrier. The concentration of DMSO in the vehicle control and the dilutions was adjusted to 1%. The fish were injected (10 ml/kg bw) with the vehicle control (1% DMSO, 25% Kolliphor in PBS), EE2 (at 10, 50 or 250 nanomole (nmol)/kg bw) or BPA (at 8, 40 or 200 micromole (μmol)/kg bw). Although these doses may not reflect environmental levels, they are within the rage of similar laboratory experiments with these compounds (51–53). The 72h sampling was based on our pilot experiments to determine optimal sampling time for gene expression assays. The fish from each group were kept in separate tanks. Sampling was performed 72 hours after injection by euthanizing the fish with a quick blow to the head. Blood was sampled in a heparinized syringe and plasma was isolated by centrifugation (1000 × g for 10 min at 4 °C) and stored at -80°C. Biometric data such as body weight, liver weight, gonadal weight, body length and sex were recorded. The liver tissue and pituitary gland were dissected and snap-frozen in liquid nitrogen and stored at -80°C until RNA extraction.

### 2.4 RNA extraction

Total RNA extraction from pituitary and liver of individual fish was performed using TRI Reagent (Sigma) according to the manufacturer’s protocols. Briefly, each frozen whole individual pituitary sample was homogenized in 250 μL TRI Reagent followed by mixing with 50 µL chloroform and centrifugation at 12000 g for 15 min. After centrifugation 125 µl of the top aqueous phase was added equal volume isopropanol and centrifuged again. The resulting RNA pellet was washed in 1 ml of 75% ethanol, dried and dissolved in 10 µL of RNAse free water. For liver, about 50 mg of individual frozen liver sample was homogenized in 1 ml TRI Reagent followed by addition of 0.2 ml chloroform, centrifugation and isopropanol precipitation of RNA from 0.5 ml of the top aqueous phase, as described above. The liver RNA pellet was dried and dissolved in 100 µL of RNAse free water. The concentration and quality of the total RNA was assessed using a NanoDrop ND-1000 spectrophotometer (NanoDrop Technologies, Wilmington, DE, USA), and the Agilent 2100 Bioanalyzer (Agilent Technologies, Palo Alto, CA).

### 2.5 RNA sequencing

The responses of all dose groups to the treatments were first assessed by qPCR for *vtg4* gene and plasma levels of Vtg protein by ELISA, which showed low but significant responses to the low dose EE2 and no significant responses to the low dose BPA group (see below). Thus, the lowest dose groups were excluded from RNA sequencing for EE2 and BPA. RNA-sequencing was performed for the female pituitary and selected liver samples from the medium dose and high dose groups of EE2 (50 and 250 nmol/kg bw) and BPA (40 and 200 μmol/kg bw). For pituitary samples, a total of 40 randomly selected female individual pituitaries (*n* = 4 per group for 40 μmol/kg bw BPA group, *n* = 6 per group for all the other medium and high dose groups) (**Supplementary Table S1A**) were submitted for RNA sequencing. Only 4 individuals were females in the 40 μmol/kg bw BPA group, as determined after dissection. For liver samples, a total of 18 samples (*n* = 3-4 per group) were analyzed from a subset of fish from each group for which pituitary samples were analyzed (**Supplementary Table S1A**).

RNA sequencing was performed at the Genomics Core Facility at the University of Bergen, as previously described (48). Sequencing library was prepared from RNA samples from individual pituitary or liver samples (0.4 μg total RNA per sample) according to Illumina TruSeq^®^ Stranded mRNA Sample Preparation Guide (Illumina, Inc., San Diego, CA, USA). Briefly, poly(A)+ RNA was purified and fragmented, and double strand cDNA synthesized followed by ligation of sequencing adapters and PCR amplification. The resulting cDNA library was sequenced on Illumina HiSeq 4000 system to generate 40-50 million 75 bp paired-end reads per sample.

### 2.6 Differential expression analysis of RNA-seq data

RNA-seq analysis was performed using the RASflow workflow (54). Reads were aligned to the most recent Atlantic cod reference genome (GadMor3) downloaded from Ensembl (Ensembl.org) using HISAT2 v2.1.0 (55). Counts were generated from the alignment using SAMtools v1.4.1 (56). A total of 46 samples were analyzed. Differential expression analysis between control and treated groups was performed for each treatment group (**Supplementary Table S1A**) from pituitary and liver tissues using edgeR v3.26.0 (57) and “TMM” normalization method. Genes with adjusted p-value < 0.05 and fold-changes of minimum 1.5 (for up-regulated) or maximum 0.67 (for downregulated) were considered as differentially expressed genes (DEGs). The RNA-seq data for the pituitary and liver samples have been deposited in the ArrayExpress database at EMBL-EBI and can be accessed at https://www.ebi.ac.uk/ena under accession numbers E-MTAB-12762 and E-MTAB-12604, respectively.

### 2.7 Pathway analysis

Pathway analysis was performed with the list of DEGs from each treatment group, as described previously (48). Briefly, Gene IDs or symbols of human and zebrafish orthologs of *G. morhua* genes retrieved from Ensembl database using the Biomart tool were used in pathway analysis using Enrichr (58) and Metascape (59) tools. Network and pathway enrichment and visualization was performed using ClueGo and Clupedia applications in Cytoscape (60–62). Default settings and thresholds of significant enrichment for multiple-testing adjusted *p*-values, *p* < 0.05 were used in pathway enrichment analyses, unless specified otherwise.

### 2.8 Hierarchical clustering

Hierarchical clustering analysis (Euclidian metric, complete linkage) was performed in Qlucore Omics Explorer (Qlucore, Lund, Sweden), using log2-transformed normalized counts.

### 2.9 Quantitative RT-PCR

Reverse transcription (RT) of pituitary and liver RNA samples (from all groups) followed by quantitative (qPCR) was performed as described before (48). Briefly, for each sample, total cellular RNA (1 µg in 20 µL reaction mix) was converted to cDNA using the iScript cDNA synthesis kit (BioRad). Quantitative PCR analyses was performed for each cDNA sample (5 µl of 1:10 dilution) using LightCycler^®^ 480 SYBR Green I Master mix (Roche Diagnostics, Mannheim, Germany) on BioRad CFX96 real-time PCR detection system (Bio-Rad Laboratories, Hercules, CA, USA). For each primer pair, standard curves were prepared from serial dilutions of pooled cDNAs of the RT reactions following standard protocols (63). Amplifications were performed with the temperature profile of 95 °C for 5 min followed by 40 cycles of 95 °C for 10 sec, 55 °C for 20 sec, 72 °C for 30 sec. The primers used are listed in **Supplementary Table S1B**. Housekeeping genes *rpl22* and *bact* were tested and found to be stable for use for pituitary (*rpll22*) and liver (both *rpl22* and *bact*) qPCR assays. Normalization and quantification were performed according to ΔΔCt method (63).

### 2.10 Plasma vitellogenin ELISA

Plasma vitellogenin (Vtg) ELISA (enzyme-linked immunosorbent assay assay) was performed using Atlantic cod Vtg ELISA kit as described in manufacturer’s instructions (Biosense Laboratories, Bergen, Norway). Vtg ELISA was performed for all female fish in the control and treated groups. The number of fish, *n = 6* for all groups except the two BPA groups (8 and 40 μmol/kg bw), for which *n = 4*.

### 2.11 Statistical analysis

Statistical analyses of biometric and morphometric data (condition factor, GSI and HSI), qPCR and ELISA assay data were performed using GraphPad Prism software V. 9. (GraphPad Software Inc., San Diego, CA). Data were log-transformed when necessary to meet the requirements for normal distribution and homogeneity of variance. Statistical tests were performed using one-way analysis of variance followed by Dunnett’s *post-hoc* test for multiple comparisons. Analyses of data with non-normal distribution were performed with non-parametric ANOVA (Kruskal-Wallis), followed by a Dunn’s test for multiple comparisons.

## 3 Results

### 3.1 Exposure doses and biometric data

No apparent toxic effects were observed for fish in any of the treatment groups. Liver weight, body weight (bw), body length (L), gonadal weight, hepatosomatic index (liver weight/bw × 100), gonado-somatic index (GSI) (gonad weight/bw × 100) and Fulton’s condition factor (100 × bw/L^3^) were determined and compared with untreated control group using one-way ANOVA in GraphPad Prism software V. 9. No significant effects were observed for any of the biometric parameters (**Supplementary Figure S1**). All fish were sexually immature with mean gonado-somatic index of 0.6, which is classified as stage I of ovarian development (32).

### 3.2 Effects in the pituitary

#### 3.2.1 Overview of differentially expressed genes in the pituitary

**Figure 1:**
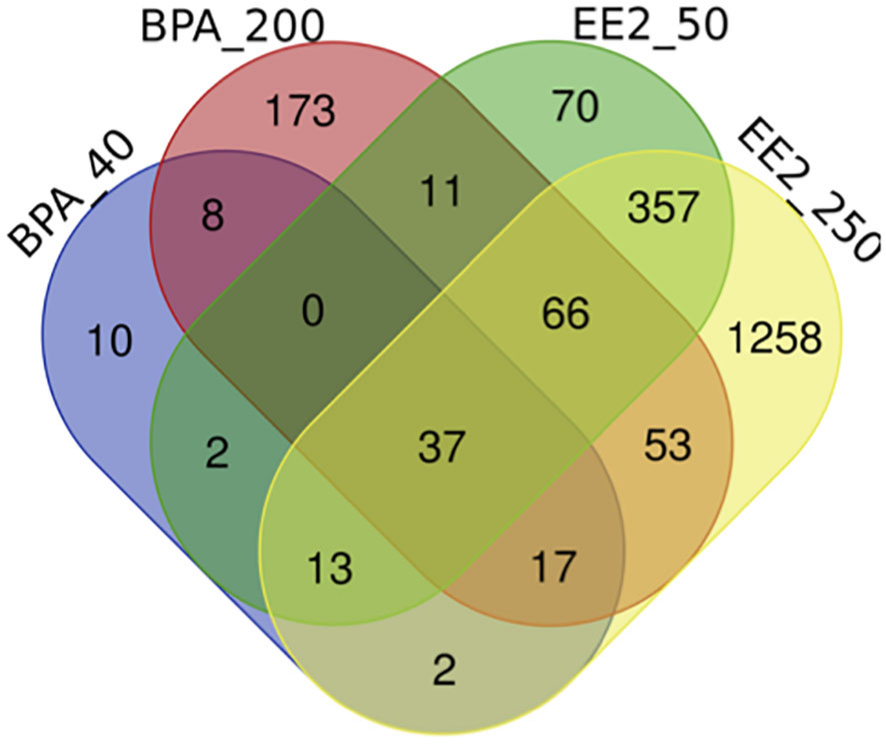
Venn diagram comparison of the number of differentially expressed genes (DEGs) in the pituitary of juvenile female Atlantic cod treated with BPA (40 or 200 μmol/kg bw) or EE2 (50 or 250 nmol/kg bw). BPA_40: BPA 40 μmol/kg bw; BPA_200: BPA 200 μmol/kg bw; EE2_50: EE2 50 nmol/kg bw; EE2_250: EE2 250 nmol/kg bw. The webtool (http://bioinformatics.psb.ugent.be/webtools/Venn/) was used to draw the Venn diagram.

The lists of pituitary DEGs from both doses of BPA (392 genes) and EE2 (1886 genes) are presented in **Supplementary Tables S2**, **S3**. The Venn diagram (**Figure 1**) shows comparison of the pituitary DEGs for all groups of BPA and EE2. BPA resulted in much lower number of DEGs than EE2. But about 50% DEGs in the BPA groups were shared with the EE2 groups (**Figure 1**).

**Figure 2:**
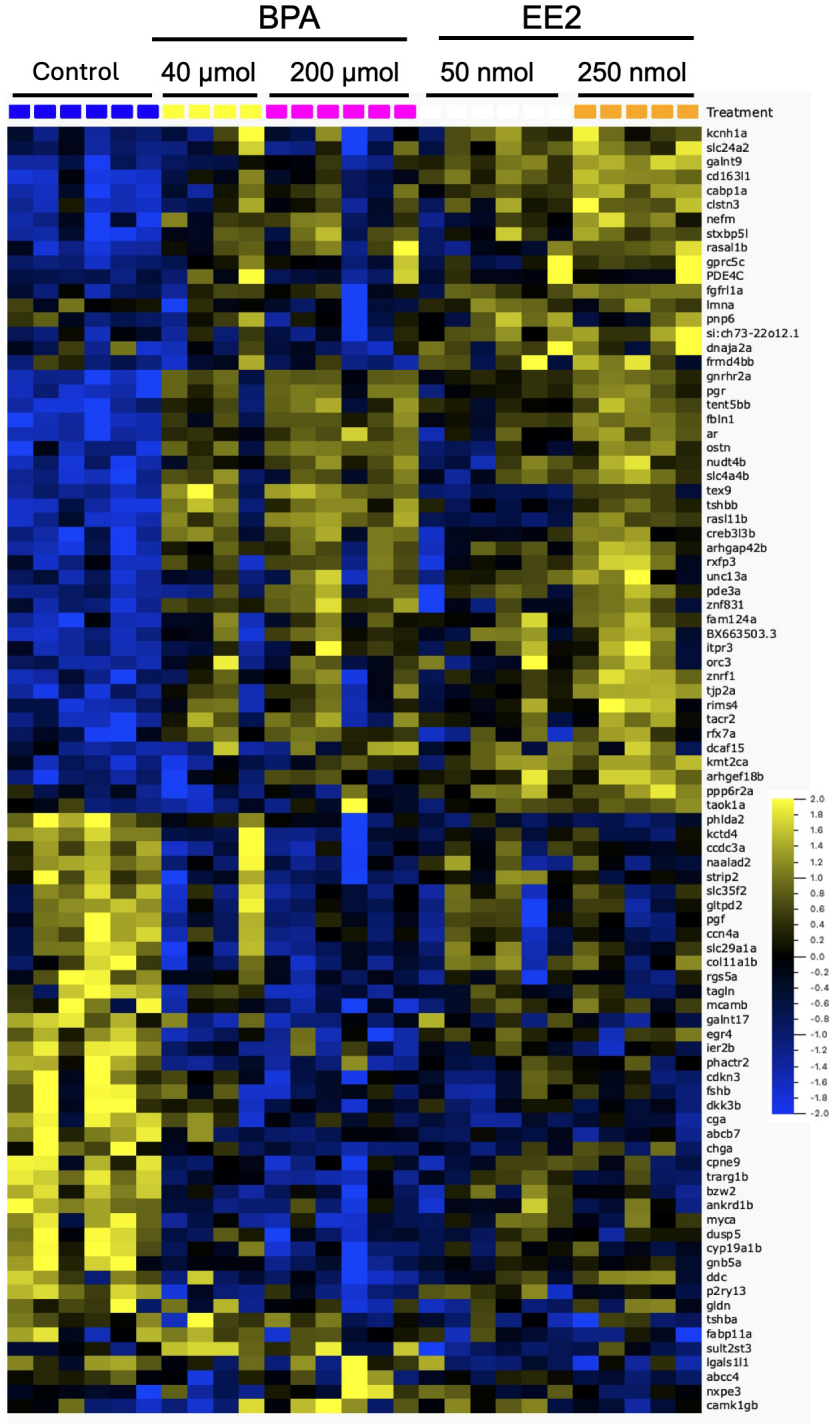
Heatmap showing the top differentially expressed genes by both BPA and EE2 in the pituitary of juvenile female Atlantic cod. The genes represent the top affected of the shared DEGs from BPA and EE2 groups (combined medium and high groups) (**Supplementary Figures S3A, B**). The doses indicated are per kg body weight. The yellow and blue ends of the color bar indicate high and low relative expression levels, respectively.

**Figure 3:**
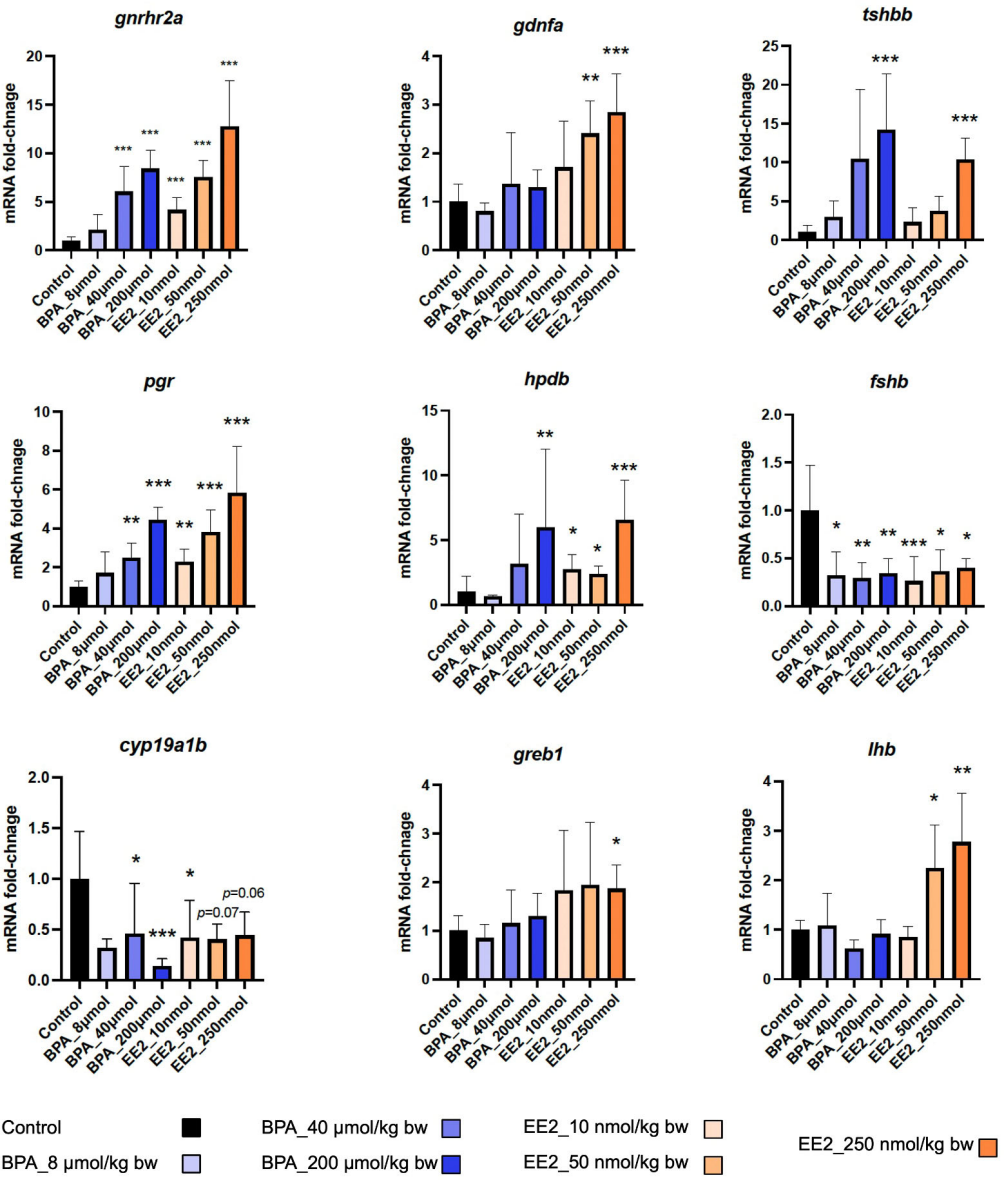
qPCR analysis of selected genes in the pituitary of juvenile female Atlantic cod exposed to indicated doses of BPA and EE2 (n = 3-6 per group). The doses indicated are per kg body weight. Data represent mean ±; sd. Asterisk (*) indicates statistically significant difference from the control group (*p < 0.05, **p < 0.01, ***p < 0.001).

Dose-response trends were assessed from expression profiles shown for the two dose groups of EE2 (**Supplementary Figure S2A**) and BPA (**Supplementary Figure S2B**). The EE2 groups showed strong dose-response trends, while such trends were less apparent for the BPA groups. The heatmap in **Figure 2** shows expression profiles of the top genes affected in both BPA and EE2 groups. RNA-seq was not performed for the low dose groups of BPA and EE2. Therefore, to assess dose-response trends in all samples including the low dose groups, some of the DEGs were also analyzed by qPCR (**Figure 3**). The qPCR assay showed generally good dose-response trends for each compound, particularly for EE2 groups (see below).

Comparison of the lists of DEGs in BPA and EE2 groups showed 201 shared genes (**Figure 1**). For comparison of enriched pathways (**Table 1**), Venn diagram comparison of the DEGs with human orthologs was constructed that showed 148 DEGs (**Supplementary Figures S3A, B**), of which top affected DEGs are shown in a heatmap (**Figure 2**). Although BPA affected much lower number of genes than EE2, many top genes and important pathways (see below) were similarly affected by both EE2 and BPA (**Figure 2**, **Table 1**, **Supplementary Figure S3B**).

**Table 1:** KEGG pathways enriched in pituitaries of juvenile female Atlantic cod treated with BPA and EE2.

Term	Adjusted P-value	Genes
cAMP signaling pathway	3.9E-03	***abcc4, grin3a, pln, creb3l3a, fshb, pde4c, pde3a*, cga*, tshba***
Neuroactive ligand-receptor interaction	3.9E-03	***p2ry13, grin3a, galr1a, fshb, chrna9, gnrhr4, tacr2, cga, adra1aa, tshba, rxfp3***
Calcium signaling pathway	4.1E-03	***fgf8a, pln, gdnfa, erbb3a, tacr2, itpr3, plcd3a, adra1aa, camk1ga***
Dopaminergic synapse	1.5E-02	***ddc, creb3l3a, gng8, itpr3, gnb5a, kcnj3a***
GnRH signaling pathway	1.5E-02	***egr1, fshb, gnrhr2a, itpr3, cga***
PI3K-Akt signaling pathway	2.1E-02	***kitlga, fgf8a, creb3l3a, erbb3a, myca, gng8, gnb5a, itgb6, pgfb***
Serotonergic synapse	2.1E-02	***ddc, gng8, itpr3, gnb5, kcnj3a***
Cholinergic synapse	2.1E-02	***creb3l3a, gng8, itpr3, gnb5a, kcnj3a***
MAPK signaling pathway	2.1E-02	***dusp5, kitlga, fgf8a, erbb3a, myca, taok1b, cacng3, pgfb***
Glutamatergic synapse	2.1E-02	***grin3a, gng8, itpr3, gnb5a, kcnj3a***
Ras signaling pathway	2.1E-02	***shc4, kitlga, fgf8a, gng8, rasal1b, gnb5a, gab***
Chemical carcinogenesis	2.3E-02	***ar, fgf8a, creb3l3a, myca, chrna9, pgr, sult1st4***
Thyroid hormone synthesis	2.5E-02	***creb3l3a, itpr3, cga, tshba***
Relaxin signaling pathway	2.7E-02	***shc3, creb3l3a, gng8, gnb5a, rxfp3***
Apelin signaling pathway	3.2E-02	***egr1, gng8, itpr3, gnb5a, prkag3b***
Estrogen signaling pathway	3.2E-02	***shc4, creb3l3a, pgr, itpr3, kcnj3a***
Hypertrophic cardiomyopathy	3.8E-02	***lmna, itgb6, cacng3, prkag3b***
Retrograde endocannabinoid signaling	3.9E-02	***ndufv1, gng8, itpr3, gnb5a, kcnj3a***
Oxytocin signaling pathway	3.9E-02	***itpr3, cacng3, prkag3b, camk1ga, kcnj3a***
Circadian entrainment	3.9E-02	***gng8, itpr3, gnb5a, kcnj3a***
Ovarian steroidogenesis	4.1E-02	***fshb, cga, cyp19a1b***
Parathyroid hormone synthesis, secretion and action	4.8E-02	***egr1, creb3l3, pde4c, itpr3***
cGMP-PKG signaling pathway	4.8E-02	***pln, creb3l3, pde3a, itpr3, adra1aa***
Tight junction	4.9E-02	***rab13, arhgef18b, prkag3b, tjp2***

KEGG pathway enrichment analysis was performed using DEGs shared between BPA (40 or 200 μmol/kg bw) and EE2 (50 or 250 nmol/kg bw) using Enrichr (58). The expression profile of the top shared DEGs is shown in **Figure 2**. The genes populating the KEGG pathways are shown in red and light green colored fonts, for upregulated and downregulated genes, respectively.

Some of the top modulated genes by BPA and EE2 are related to known pituitary hormones involved in reproductive and metabolic homeostatic processes (**Figure 2**, **Supplementary Tables S2**, **S3**). These include genes encoding nearly all the subunits of pituitary glycoprotein hormones (Cga), follicle stimulating hormone (Fshb), and thyroid stimulating hormones (Tshbb and Tshba)). The *fshb*, *cga* (encoding the common alpha subunit of Fsh, Lh and Tsh) and *tshba* genes were downregulated in the EE2 and BPA treated groups (**Figure 2**). Interestingly, a second variant of *tshb* gene (*tshbb*) was upregulated (**Figure 2**). The luteinizing hormone beta subunit gene (*lhb*) (not significantly affected in the RNA-seq analysis) was also found to be significantly upregulated by EE2 in qPCR analysis (**Figure 3**). The *gonadotropin releasing hormone receptor 2a* (*gnrhr2a*) was strongly upregulated by both compounds (**Figure 2**). Other hormone related genes affected include*, proopiomelanocortin a* (*pomca*) (downregulated by EE2), and the nuclear hormone receptors genes *progesterone receptor* (*pgr*), *androgen receptor (ar)* and *mineralocorticoid receptor* (*nr3c2*), which were all up-regulated (**Figure 2**, **Supplementary Tables S2**, **S3**). In addition, the *parathyroid hormone-like hormone a* (*pthlha*) and the brain *aromatase cytochrome p45019a1b* (*cyp19a1b*) genes were affected by BPA and EE2 (**Figure 2**, **Supplementary Tables S2**, **S3**). Examples of strongly upregulated signaling and growth factor-related genes include *prkag3b*, *gng8 cabp1a*, *creb3l3b, greb1* and *gdnfa* (**Figure 2**, **Supplementary Tables S2**, **S3**).

#### 3.2.2 Pathways affected in the pituitary

**Figure 4:**
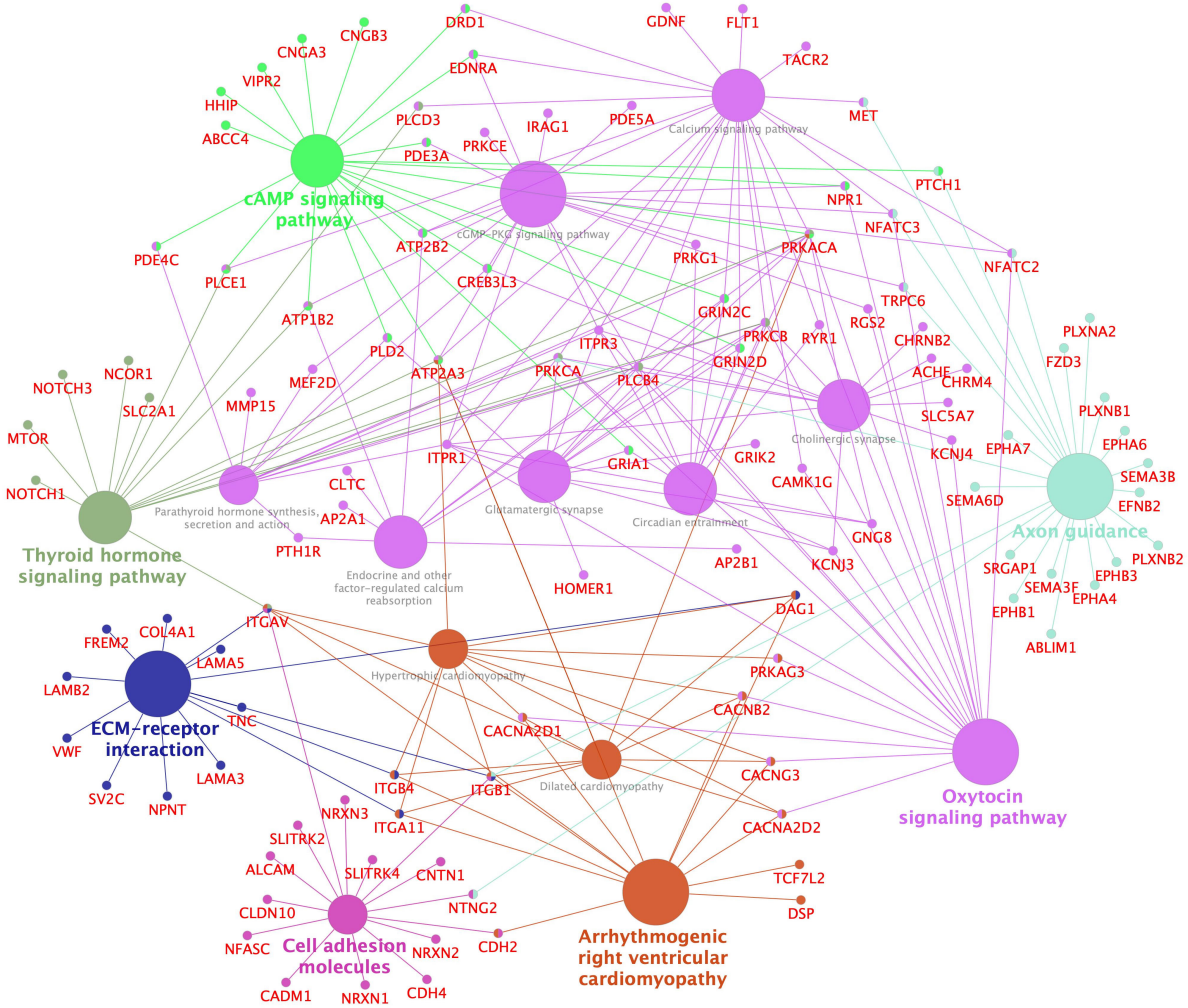
Enriched KEGG pathways and networks in upregulated genes in pituitaries of ethynylestradiol (250 nmol/kg body weight) treated juvenile female Atlantic cod. Network enrichment and visualization was performed using Cytoscape ClueGo application. Only significantly enriched (adjusted *p*-value < 0.05) KEGG pathways are shown. The size of a pathway term node is proportional to the number of populating genes. The pathway term nodes belonging to similar pathways share same color and the most connected pathway term is in bold fonts. Gene name nodes and edges share the color of pathways term nodes they are connected to. The whole list of enriched KEGG terms (for both up- and downregulated genes) are shown **Supplementary Figures S5A–D**.

To have an overview of the major affected function, pathway and network enrichment analysis was performed using Enrichr (58), Metascape (59) and Cytoscape (62). More detailed pathway analysis was performed for the high dose groups and comparisons were made between the dose groups, as well as between BPA and EE2, as presented below. Pathways enriched in the genes upregulated by the high dose EE2 group were visualized in Cystoscape (**Figure 4**). Some of the top pathways enriched in the upregulated genes are related to pituitary reproductive hormones, metabolic homeostatic hormones (thyroid and parathyroid) as well as neurotransmitter related processes. In addition, cell signaling, growth and differentiation related pathways were enriched in the high dose EE2 group (**Figure 4**). Similar pathway and network enrichment analysis in the downregulated genes showed that the most predominant KEGG pathways were related to more general cellular processes such as RNA and protein metabolism, and oxidative phosphorylation (**Supplementary Figure S4**). Genes and pathways related to neurotransmitter functions such as biogenic amine metabolism including the genes *dbh* and *ddc* (**Figure 2**, **Supplementary Tables S2**, **S3**) were also affected in the pituitary exposed to the estrogenic compounds (**Figure 4**, **Supplementary Figures S5A–D**). Notably, a gene encoding the enzyme 4-hydroxyphenylpyruvate dioxygenase (Hpdb) that is involved in tyrosine and dopamine metabolism pathway was induced by BPA and EE2 in the pituitary (**Figure 3**). In parallel, a paralog of the *hpdb* gene, *hpdl* was also induced by both BPA and EE2 in the liver (**Supplementary Tables S4**, **S5**).

Pathways enriched in the two dose groups of each compound were also compared after KEGG pathway and GO biological process enrichment analyses (**Supplementary Figures S5A–D**, **S6**). For EE2 group, comparison of the two doses suggests dose-response effects with similar affected pathways although many pathways were not significantly enriched in the lower dose group (**Supplementary Figures S5A, B**). For BPA, only few KEGG pathways were significantly enriched at each dose, but some pathways were shared by the two dose groups at less stringent enrichment threshold (**Supplementary Figures S5C, D**). Similarly, enriched Gene Ontology (GO) terms show similar trends, with the medium dose sharing some pathway terms with the high dose (**Supplementary Figure S6**).

#### 3.2.3 Comparison of pituitary effects of BPA and EE2

To compare BPA and EE2, enrichment analysis was performed for 148 DEGs shared between the two compounds (**Supplementary Figures S3A, B**), using Enrichr (58). **Table 1** shows that BPA shares many top pathways affected by EE2. So, although fewer genes were affected in BPA groups, many key genes such as genes encoding pituitary glycoproteins and other peptide hormones as well as various signaling pathways were shared with the EE2 groups (**Table 1**, **Supplementary Figure S3B**). The heatmap (**Figure 2**) shows the expression profile of the top DEGs shared by EE2 and BPA.

We also looked at the pathways enriched in the genes exclusively modulated by BPA and EE2. There were 130 and 1171 DEGs specific for BPA and EE2 groups, respectively (**Supplementary Figure S3A**). No KEGG pathways were significantly enriched in the 130 DEGs specific for BPA. The KEGG pathways significantly enriched in the EE2 specific DEGs (**Supplementary Figure S3C**) are very similar to the list of KEGG pathways enriched in high dose of EE2 (**Supplementary Figure S5A**). Overall, the pathways that appear to be most relevant to pituitary functions such as glycoprotein hormone related processes are largely represented in the shared pathways shown in **Table 1**.

### 3.3 Effects in the liver

#### 3.3.1 Overview of differentially expressed genes in the liver

**Figure 5:**
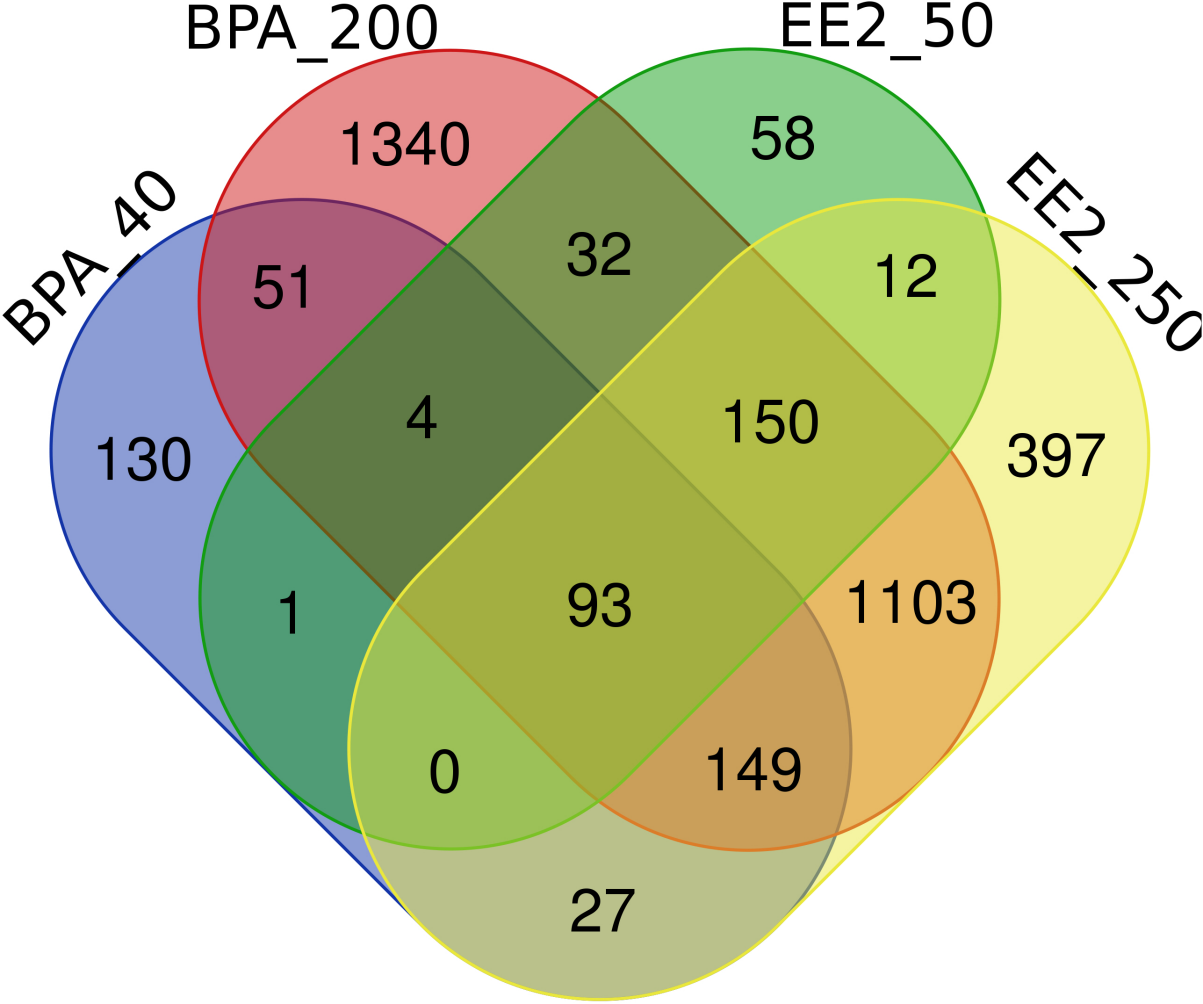
Venn diagram comparison of the number of differentially expressed genes (DEGs) in the liver of juvenile female Atlantic cod treated with BPA (40 or 200 μmol/kg bw) or EE2 (50 or 250 nmol/kg bw). BPA_40: BPA 40 μmol/kg bw; BPA_200: BPA 200 μmol/kg bw; EE2_50: EE2 50 nmol/kg bw; EE2_250: EE2 250 nmol/kg bw. The Venn diagram was drawn using the webtool at http://bioinformatics.psb.ugent.be/webtools/Venn/.

In the liver, a total of 3080 DEGs were detected in the BPA groups (**Supplementary Table S4**). For EE2 groups, a lower number (2026 DEGs) was detected (**Supplementary Table S5**). This contrasts with the effects in the pituitary, where EE2 affected much higher number of DEGs (**Figure 1**). In the liver, the two compounds showed high similarities in their effects on gene expression, as can be seen from the Venn diagram (**Figure 5**) and heatmap (**Supplementary Figure S7A**). Similarities in all the treatment groups are observed in the expression profiles (**Supplementary Figure S7A**) and heatmap of shared pathways (**Supplementary Figure S7B**). Dose-response trends can also be observed for the expression profiles of many top genes in the two dose groups of each compound (**Supplementary Figure S7A**). Dose-response trends were also evaluated for all the three dose-groups of the compounds including the low dose, using qPCR and Vtg ELISA assays, which showed generally good dose-response trends and good concordance with the RNA-seq data (**Supplementary Figures S8A–D**).

As expected, several well-known estrogen-regulated genes encoding proteins associated with vitellogenesis related process such as the estrogen receptor 1 (Esr1), vitellogenins (Vtgs) and eggshell zona pellucida (Zp) proteins were the most strongly up-regulated by the xenoestrogens (**Supplementary Tables S4**, **S5**). Apart from these estrogen marker genes that were upregulated, the DEGs list is highlighted by many lipid metabolism related genes that were downregulated in a coordinated manner by both compounds (**Supplementary Tables S4**, **S5**), as described below.

#### 3.3.2 Pathways affected in the liver

To compare pathways perturbed in each group, pathway enrichment analysis was performed for the downregulated (**Supplementary Figures S9A–D**) and upregulated (**Supplementary Figures S10A, B**) set of genes, using Enrichr (58). Many of the enriched top 20 pathways in the downregulated genes from BPA and EE2 groups are related to energy metabolism, mainly lipid metabolism, particularly in the high dose groups (**Supplementary Figures S9A–D**). In the lists of the upregulated genes in the high dose groups of BPA and EE2, few KEGG pathways were significantly enriched, and those are related to general cellular processes such as RNA and protein processing (**Supplementary Figures S10A, B**). However, we note the strong upregulation of many estrogen marker genes (**Supplementary Tables S4**, **S5**) related to fish specific oogenesis pathway. Similar analysis did not result in significantly enriched KEGG pathways in the upregulated genes from medium dose groups of BPA and EE2.

**Figure 6:**
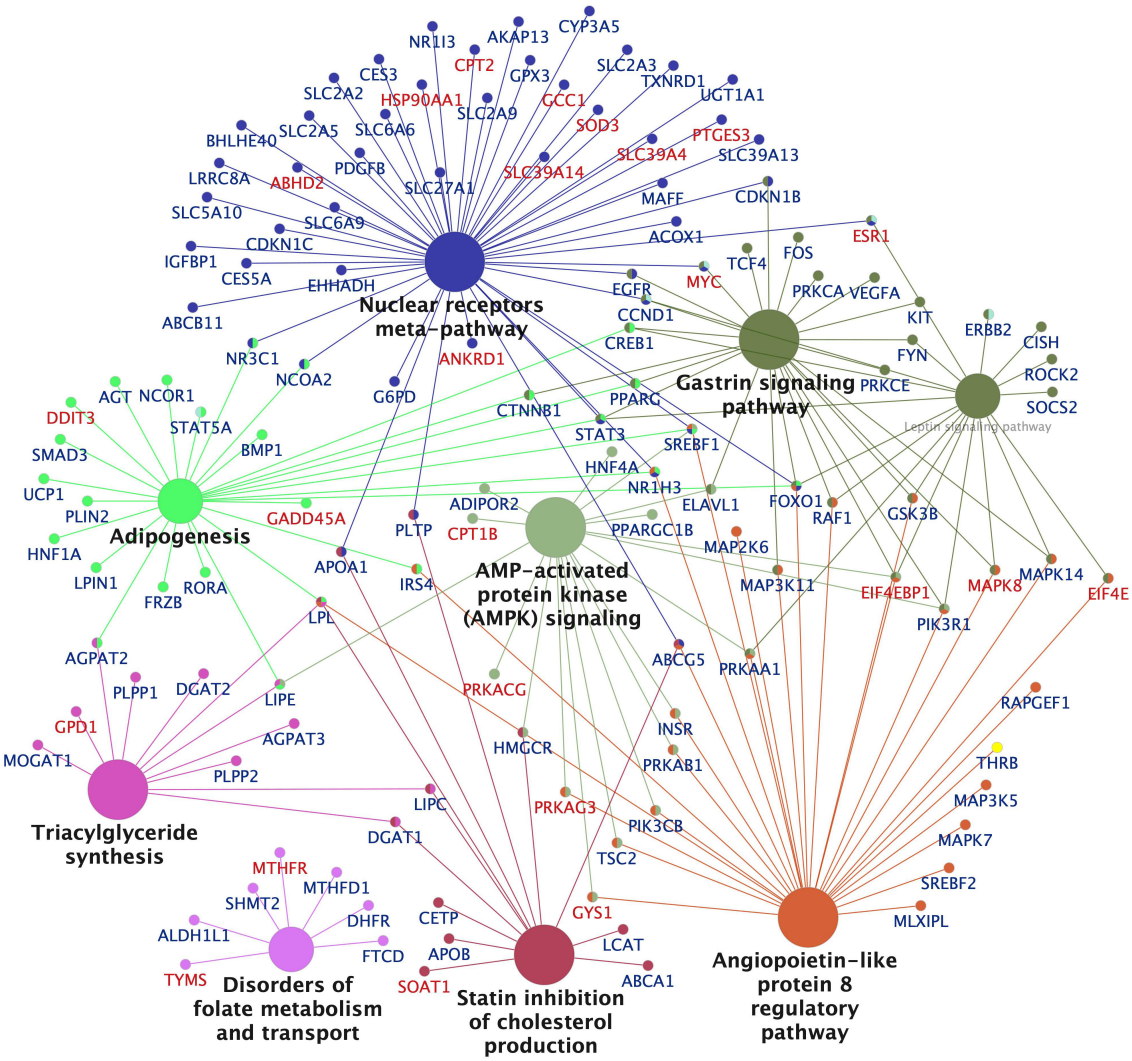
Network visualization of significantly enriched KEGG pathways and populating differentially expressed genes in the liver of Atlantic cod treated with ethynylestradiol (250 nmol/kg body weight). Significantly enriched networks and KEGG pathways generated in Cytoscape using the ClueGo application are shown. The size of a pathway node is proportional to the number populating genes. The pathway term nodes belonging to similar pathways share same color, and the most important term is in bold face. The symbols of upregulated and downregulated genes are colored red and blue, respectively. A larger network visualization of the enrichment analysis shown in the supplementary file (**Supplementary Figure S11**).

The Cytoscape network and pathway analysis tool was used to visualize enriched pathways and the populating DEGs for the high dose groups of EE2 (**Figure 6**, **Supplementary Figure S11**) and BPA (**Supplementary Figure S12**). The networks show that the top pathways affected in the EE2 and BPA treatment groups were similar and dominated by lipid metabolism pathways such as LXR (liver X receptor) and PPAR (peroxisome proliferator-activated receptor) signaling, metabolism of fatty acids, triglycerides, glycerophospholipids, cholesterol, plasma lipoprotein assembly, lipid transport as well as carbohydrate metabolism, including genes encoding glucose transporters (e.g., *slc2a2* and *slc25a*, downregulated) and glycolytic enzymes (e.g., *pfkfb1*, upregulated) (**Figure 6**, **Supplementary Figures S11**, **S12**). Other top pathways affected, particularly in the high dose exposed groups of EE2 and BPA include pathways and genes related to protein synthesis and rRNA processing (upregulated), intra-Golgi and Golgi to ER retrograde transport (mostly upregulated) (**Supplementary Figure S11**). As observed with the KEGG pathway analyses of downregulated genes (**Supplementary Figures S8A-D**), most of the genes populating lipid synthesis pathways in the EE2 and BPA groups were downregulated. For example, several lipogenesis promoting genes such as *nr1h3* (*lxra*), *pparg*, *srebf1*, *srebf2*, *lpin1, acly, gpat3 dgat1*, and *dgat2* were downregulated (**Supplementary Tables S4**, **S5**, **Figure 6**, **Supplementary Figures S11**, **S12**). In parallel, some genes in the mammalian PPARA pathway that promote lipid catabolism such as *cpt1a*, *cpt1b*, *cpt2*, *acads*, *acsl1*, and *acaa2* were upregulated (**Supplementary Tables S4**, **S5**, **Figure 6**, **Supplementary Figures S11**, **S12**). Furthermore, in the cholesterol metabolism pathway, the *hmgcr* gene encoding the rate-limiting enzyme for cholesterol biosynthesis and the gene encoding for its esterification, *soat1*, were strongly downregulated and upregulated, respectively (**Supplementary Figures S11**, **S12**).

#### 3.3.3 Comparison of pathways affected by BPA and EE2 in the liver

Pathways affected by BPA and EE2 were compared by performing pathways enrichment analysis in the liver DEGs shared and exclusive to each compound. The Venn diagram in **Supplementary Figure S13A** shows 1214 shared DEGs. The figure also shows 1089 and 304 DEGs specific for BPA and EE2 groups, respectively. Some KEGG pathways were significantly enriched in the list of DEGs specific for BPA (**Supplementary Figure S13B**). Only one KEGG pathway was significantly enriched in the EE2 specific DEGs (**Supplementary Figure S13C**). Several KEGG dominated by energy pathways as described above were significantly enriched in the list of shared DEGs (**Supplementary Figure S13D**). Many of the pathways enriched in the list of DEGs specific for BPA are very similar to the list of KEGG pathways enriched in the shared DEGs (**Supplementary Figure S13D**) and DEGs in the high dose of EE2 (**Supplementary Figure S9A**). Therefore, there does not seem to be notable differences between pathways affected by BPA and EE2 in the liver.

### 3.4 qPCR analysis

RNA-seq analysis was used only for the control, medium and high dose treatment groups. qPCR analysis was performed to assess dose-response trends using all groups including the low dose groups. Eight genes were selected from the list of pituitary DEGs including some of the top affected key genes which were included to further evaluate their potential as biomarkers. The *lhb* gene not in the DEGs list was also included because it was previously shown to be induced by estrogens and xenoestrogens (64, 65). For liver, six DEGs were tested by qPCR. The qPCR assays were also used to confirm the RNA-seq data. As shown in **Figure 3** the expression levels for many of the genes show good dose-response trends in the pituitary. All the genes were significantly differentially expressed (compared to the controls) in at least one EE2 group. For the BPA groups that showed generally weaker dose-response trends, two of the genes (*greb1* and *lhb*) were not significantly affected (**Figure 3**). The *lhb* gene was not significant in the RNA-seq analysis but the qPCR assay showed significant upregulation in the EE2 groups (**Figure 3**). The qPCR assay for the pituitary DEGs show good concordance with the RNA-seq results (**Figure 3**). Similarly, in the liver, the selected genes also showed significant changes for at least one dose of each compound except for *pparg* (for BPA and EE2) and *cyp46a1.1* (for BPA) (**Supplementary Figure S8A**) and qPCR results were consistent with the plasma vitellogenin ELISA assay (**Supplementary Figure S8B**). Expression fold-changes obtained using RNA-seq and qPCR assays were compared, and the two methods show good concordance for most of the pituitary (**Supplementary Figure S8C**) and liver DEGs (**Supplementary Figure S8D**).

### 3.5 Effects on plasma vitellogenin levels

Vitellogenin ELISA was performed for control and all groups treated with different doses of the estrogenic compounds (**Supplementary Figure S8B**). The ELISA assay showed significant increases in plasma vitellogenin levels in all treated groups except in the lowest dose group of BPA (8 µmol/kg bw). Similar changes of vitellogenin transcript levels were observed in the liver of all groups, as determined by qPCR assay (**Supplementary Figure S8A**).

## 4 Discussion

In this study, we performed a comprehensive mapping of the pituitary and liver transcriptome of Atlantic cod responding to the xenoestrogens EE2 and BPA. Our analysis suggested that BPA and EE2 affect the expression of genes in a broad range of pathways in both the pituitary and liver. Overall comparison of the two compounds shows that pituitary effects of BPA appeared generally weaker, although many of the top enriched genes and pathways were shared with the EE2 groups. In the liver, BPA and EE2 generally resulted in similar effects on most of the top affected estrogen target genes and pathways. These apparent differences in BPA and EE2 effects in the two organs might be attributed to differences in pharmacokinetic properties of the compounds. BPA has a shorter half-life of 3.75h (51) than EE2, which has a half-life of 20-132h (24). In both tissues, the two compounds appear to modulate multiple pathways not only in reproductive processes but also in metabolic functions as discussed below.

### 4.1 Potential reproductive and metabolic disruption by BPA and EE2 in the pituitary

Many of the enriched pathways related to cell signaling, differentiation and endocrine hormone functions appear to be related to perturbations of the synthesis and secretion of the pituitary glycoprotein hormone genes. These effects were generally shared by the high dose groups of BPA and EE2. Estrogens are well known to affect pituitary gene expression of gonadotropins and related hormones (64–67) and these effects are known to involve significant remodeling of the pituitary involving growth and differentiation of cell types (3). Perturbations of multiple pathways observed here by BPA and EE2 appear to be consistent with these complex processes regulated by estrogens. Some of the signaling pathways enriched here such as cAMP, dopamine and calcium signaling pathways are known to be involved in the synthesis and secretion of the glycoprotein hormones (68, 69). Neuroendocrine control of gonadotropins involves brain signals, mainly Gnrh that through activation of Gnrhr, regulates the synthesis of gonadotropins that control gonadal growth and steroidogenesis (4, 70). Estrogens exert feedback control in the brain and pituitary by regulating the synthesis of gonadotropins and this process involves a complex interaction of many factors and signaling pathways including dopamine signaling (4, 70, 71). Therefore, the modulatory effects observed here on pathways related to pituitary gonadotropin functions, suggest the potential of the BPA and EE2 to disrupt reproductive functions.

Effects on the thyroid hormone synthesis pathway by EE2 and BPA suggests a role of thyroid hormone in the reproductive pathway, which appears to be consistent with previous studies suggesting effects of estradiol on thyroid hormone levels in Atlantic cod (72). Enrichment of the calcium homeostasis and parathyroid hormone pathway in the pituitary is possibly related to calcium mobilization due to increased hepatic synthesis of vitellogenin, which contains a large amount of calcium (1, 2, 33). The pituitary gland is one of the main sources of hypercalcemic PTH-like peptides (73). Indeed, estrogen-induced vitellogenin synthesis was shown to increase calcium mobilization from scales and bones in rainbow trout (74). The effects observed on the pathways related to thyroid and parathyroid systems by BPA and EE2 therefore suggests potential effect of xenoestrogens in disrupting metabolic homeostasis.

Many processes that appear to be affected by EE2 and BPA in the current study including pathways related to Gnrh and thyroid hormone signaling, circadian rhythm and cell cycle were also previously reported to be affected by EE2 in coho salmon pituitaries (64). Our results are also corroborated by previous studies reporting up-regulation of the *prg*, *ar and gnrhr* variant genes in the pituitary of estrogen treated or sexually maturing fish (75).

The pathways related to neurotransmitter metabolism, particularly the induction of the *hpdb* gene involved in catabolism of tyrosine (76) by BPA and EE2 in the pituitary might be related to effects of estrogens on dopamine signaling in fish (3). In mammals, HPD is the key enzyme in tyrosine catabolism which is an important precursor of neurotransmitters such as dopamine (77). In addition, the paralog gene, *hpdl* (4-hydroxyphenylpyruvate dioxygenase-like) was also up-regulated by BPA and EE2 in the liver (**Supplementary Tables S4**, **S5**). These two genes are associated with neurological diseases in humans (78). The HPD enzyme has also been shown to be upregulated in the liver of BPA exposed rats (79), consistent with our results. Whether Hpd and Hpdl enzymes are involved in biotransformation of BPA and estrogens needs to be further investigated.

### 4.2 Potential perturbation of lipid metabolism by BPA and EE2 in the liver

In the liver, in addition to the classic estrogen marker genes, a more diverse set of genes and pathways were affected by the estrogenic compounds. Of these, the most dominant metabolic effects were on lipid metabolism pathways, which appear related to energy mobilization during vitellogenesis. These effects were highlighted by downregulation of key genes known to be involved in the mammalian lipogenic pathways such as genes encoding the transcription factors Srebp and Lxr and nearly all enzymes in the synthesis of triglycerides (80). The remarkably coordinated downregulation of genes in the triglyceride synthesis pathway suggests that the compounds induce a metabolic switch from lipid synthesis and storage in the liver to lipid mobilization for transport to the ovary. Some of the effects on lipid metabolism related pathways illustrated here have also been observed before in other studies in fish liver tissues and primary hepatocytes exposed to estrogenic compounds (29, 30, 81). In particular, EE2 resulted in downregulation of lipid metabolism genes in fathead minnow (29) and the authors suggested that this was related to lipid transport to the growing oocytes. Our results agree with this study and further expand the repertoire of target genes enhancing our mechanistic understanding of how exposure to xenoestrogens might disrupt energy pathways in the liver of fish. These observed transcriptional changes underlying lipid mobilization are supported by previous studies that have shown lipid mobilization as increased plasma lipid levels in estrogen-treated fish (35–37), which suggests that xenoestrogens such BPA can potentially disrupt energy pathways by mimicking estrogens. Potentially, such effects by xenoestrogens may result in untimely energy depletion in the liver that may have adverse reproductive consequences. However, weather environmental levels of xenoestrogens can modulate the energy pathways with significant effects on fish reproduction needs to be further investigated. The transfer of energy reserves stored as liver lipids to the ovaries during vitellogenesis is considered an important feature of a reproductive strategy in cod (31, 32). The effects in the liver observed here and by others using EE2 exposure (29) also illustrate the transcriptional processes underlying energy mobilization in fish liver during vitellogenesis, enhancing our understanding of the reproductive physiology.

Parallel to downregulation of lipid synthesis genes, induction of some lipid catabolism related PPARA pathway genes such as *cpt1b*, *cpt2* and *crot* was also observed here, which may represent responses to increased energy demand for the vitellogenesis related processes. Similarly, the up-regulation of the protein synthesis pathways observed might be a response to the increased demand to synthesize large amounts of egg-proteins stimulated by the estrogenic compounds (1, 2, 27, 33), also observed here as increased plasma Vtg levels. Many of the metabolism related pathways that were affected in the liver of BPA and EE2 treated fish appear to be related to the induced vitellogenesis-like process that involves massive synthesis and transport of nutrients and vitellogenin for transport to the growing oocytes.

### 4.3 Summary

In summary, a broad range of pathways related to reproductive processes and metabolic homeostatic functions of the pituitary were affected by the estrogenic compounds in this experiment. Many of the perturbed pathways appear to reflect the changes observed on expression of the genes encoding pituitary glycoprotein hormones and these effects were generally shared by the high dose groups of BPA and EE2, suggesting their similar mode of actions. In the liver, besides the well-established estrogen markers, alterations in many pathways were observed, particularly lipid metabolism that appears to mimic liver lipid mobilization during vitellogenesis stages of reproduction. We note that our study here was performed to identify estrogen target genes primarily aimed at mechanistic investigations. Therefore, the relatively high doses used here are not expected to reflect environmental levels of BPA and EE2. Further studies are needed to assess if some the target genes and pathways identified here are perturbed at low environmental levels of xenoestrogens, and to link potential effects to apical adverse outcome (82).

Finally, our results suggest that some of the strongly induced genes such *gnrhr2a*, *pgr*, *ar* and *tshbb* can be potential biomarkers to assess xenoestrogen exposure and effects in the pituitary. The *lhb* gene has been shown to be strongly induced by estrogens and xenoestrogens in salmonids and may be used as biomarker of estrogenic effects (64, 65). The *lhb* gene was not affected by BPA and weak induction in the EE2 treated fish was observed in this experiment, as shown by qPCR assay. Gonadotropin gene expression in response to estrogens may depend on species, sex and maturation stages of the fish (3, 67). Therefore, assessing multiple biomarker genes such as those suggested here may offer more robust assessment estrogenic effects in the pituitary compared to single biomarker studies.

## 5 Conclusions

Our transcriptome analysis revealed that the estrogenic compounds EE2 and BPA affect many genes involved in diverse physiological functions of the pituitary including synthesis and release of gonadotropins and thyroid hormones, suggesting potential disruption of both reproductive and homeostatic functions of the pituitary by the compounds. In the liver, the coordinated effects on genes and pathways involved in lipid metabolism reveal the transcriptome changes underlying liver lipid mobilization related to vitellogenesis, and the potential of BPA and EE2 to disrupt these processes. These results suggest that xenoestrogens can potentially perturb not only reproductive functions but also metabolic homeostatic processes in the pituitary and liver. The pituitary estrogen target genes identified here may serve as a panel of potential biomarkers of estrogenic effects.

## Data Availability

The datasets presented in this study can be found in online repositories. The names of the repository/repositories and accession number(s) can be found below: https://www.ebi.ac.uk/ena, E-MTAB-12762, E-MTAB-12604.
