# Supplementary material for: Transcriptome analysis reveals effects of ethynylestradiol and bisphenol A on multiple endocrine and metabolic pathways in the pituitary and liver of female Atlantic cod (Gadus morhua)
Source: Front Endocrinol (Lausanne). 2025 Jan 27;15:1491432. doi: 10.3389/fendo.2024.1491432 (PMC11808150; doi:10.3389/fendo.2024.1491432)
Supplement: Supplementary file 1 [file Table1.docx]

**Table S1A** Number of replicates for pituitary and liver RNA-seq analyses and the number of differentially expressed genes in each treatment group

| Compound | Vehicle | BPA | | EE2 | |
| --- | --- | --- | --- | --- | --- |
| Group | Control | BPA_40μmole | BPA_200 | EE2_50nmole | EE2_250nmole^*^ |
| Dose (μmole /kg bw) | - | 40 | 40 | 0.05 | 0.05 |
| Pituitary RNA-seq (n) | 6 | 4 | 6 | 6 | 6 |
| Pituitary DEGs |  | 89 | 365 | 556 | 1803 |
| Liver RNA-seq (n) | 4 | 3 | 3 | 4 | 4 |
| Liver DEGs |  | 455 | 2922 | 350 | 1931 |

^*^One outlier RNA-seq sample (from EE2_250nmole group) was excluded from differential expression analysis. BPA: bisphenol A; EE2: ethynylestradiol; bw: body weight; DEGs: differentially expressed genes.

**Table S1B**. Sequences of primers used in qPCR assay.
