# Supplementary material for: Transcriptome analysis reveals effects of ethynylestradiol and bisphenol A on multiple endocrine and metabolic pathways in the pituitary and liver of female Atlantic cod (Gadus morhua)
Source: Front Endocrinol (Lausanne). 2025 Jan 27;15:1491432. doi: 10.3389/fendo.2024.1491432 (PMC11808150; doi:10.3389/fendo.2024.1491432)
Supplement: Supplementary file 6 [file DataSheet1.docx]

Supplementary Figures


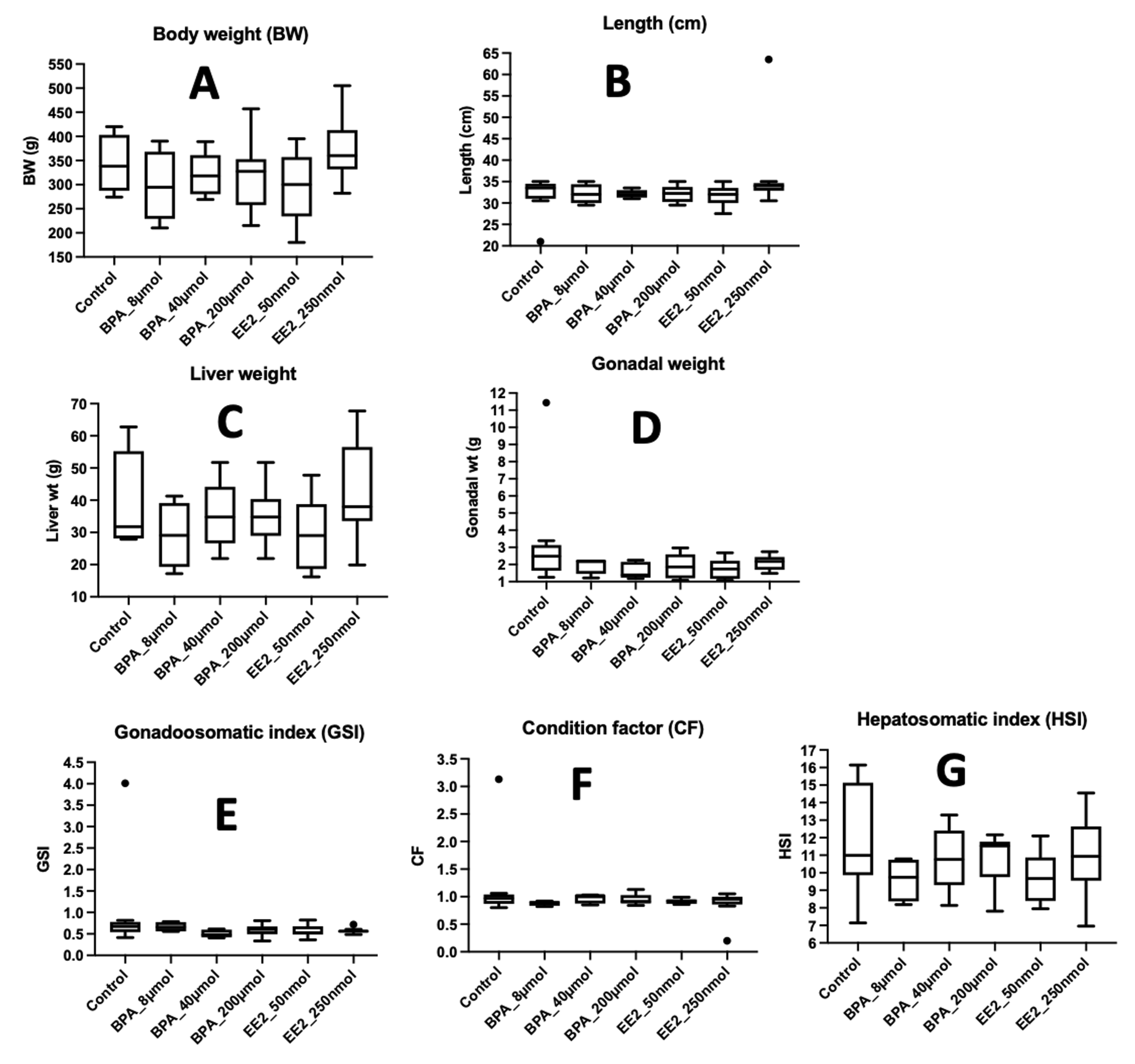


**Figure S1.** A plot o Biometric data for control and indicated dose groups of BPA and EE2 treated cod. For each group, body weight (bw) (**A**), body length (**B**), liver weight (**C**), gonadal weight (**D**), gonado-somatic index (**E**), and condition factor (**F**), and hepatosomatic index (**G**) was compared with untreated control group using one-way ANOVA in GraphPad Prism software V. 9. (GraphPad Software Inc., San Diego, CA). The different groups (x-axis) represent treatment compound and dose per kg of body weight. No significant difference was found in any of the parameters. Data presented as plot box and whiskers plot using Tukey method. n = 4-9

**
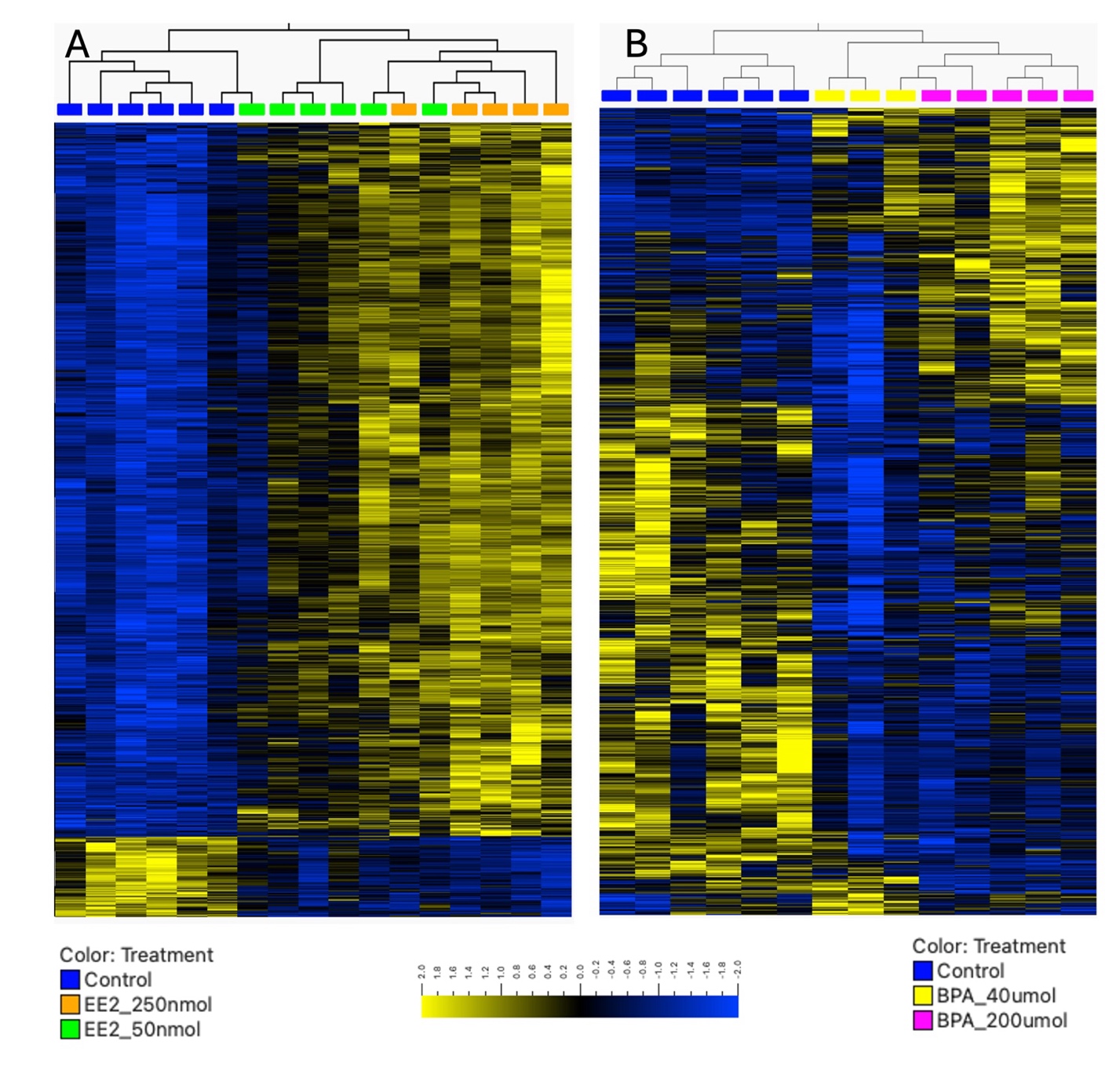
**

**Figure S2.** Heatmap of top differentially expressed genes in the pituitary of juvenile female Atlantic cod exposed to medium and high doses of EE2 (**A**) and BPA (**B**). Hierarchical clustering analysis was performed using normalized log-transformed RNA-seq read counts. The doses indicated are per kg body weight. The yellow and blue ends of the color bar (bottom) indicate high and low relative expression levels, respectively.


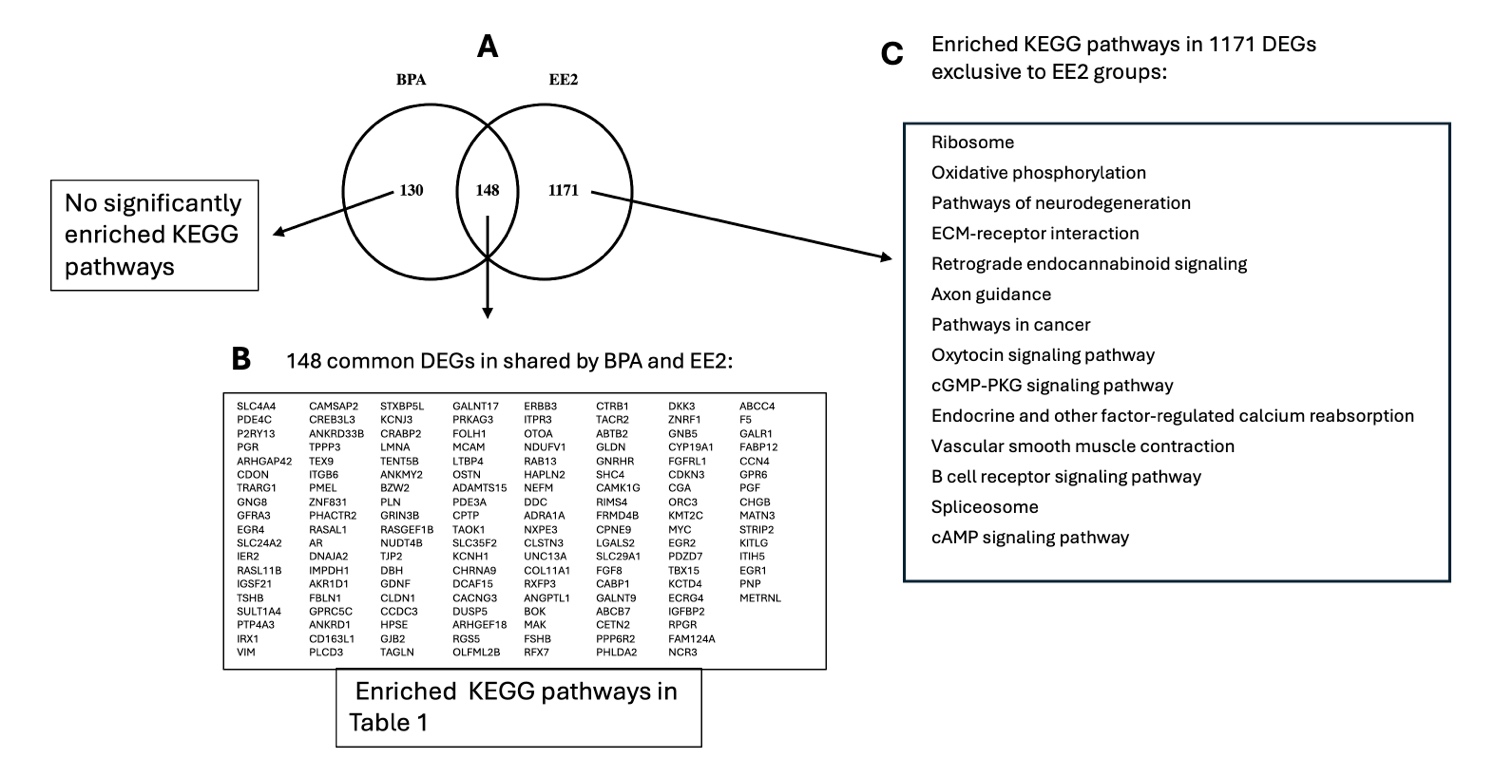


**Figure S3.** Venn diagram comparison of the lists of DEGs (with human orthologs) in BPA and EE2 groups (**A**) showing 148 shared genes (**B**). Significantly enriched KEGG pathways in the list exclusive to EE2 are terms (**C**). No significantly enriched KEGG pathways were detected in the list of 130 genes exclusive to BPA (**A**) and the enriched KEGG pathways in the list of 148 shared genes are presented in Table 1. Note that DEGs without human orthologs are not included in the comparisons. Enrichment analysis was performed in Enrichr (https://maayanlab.cloud/Enrichr/).


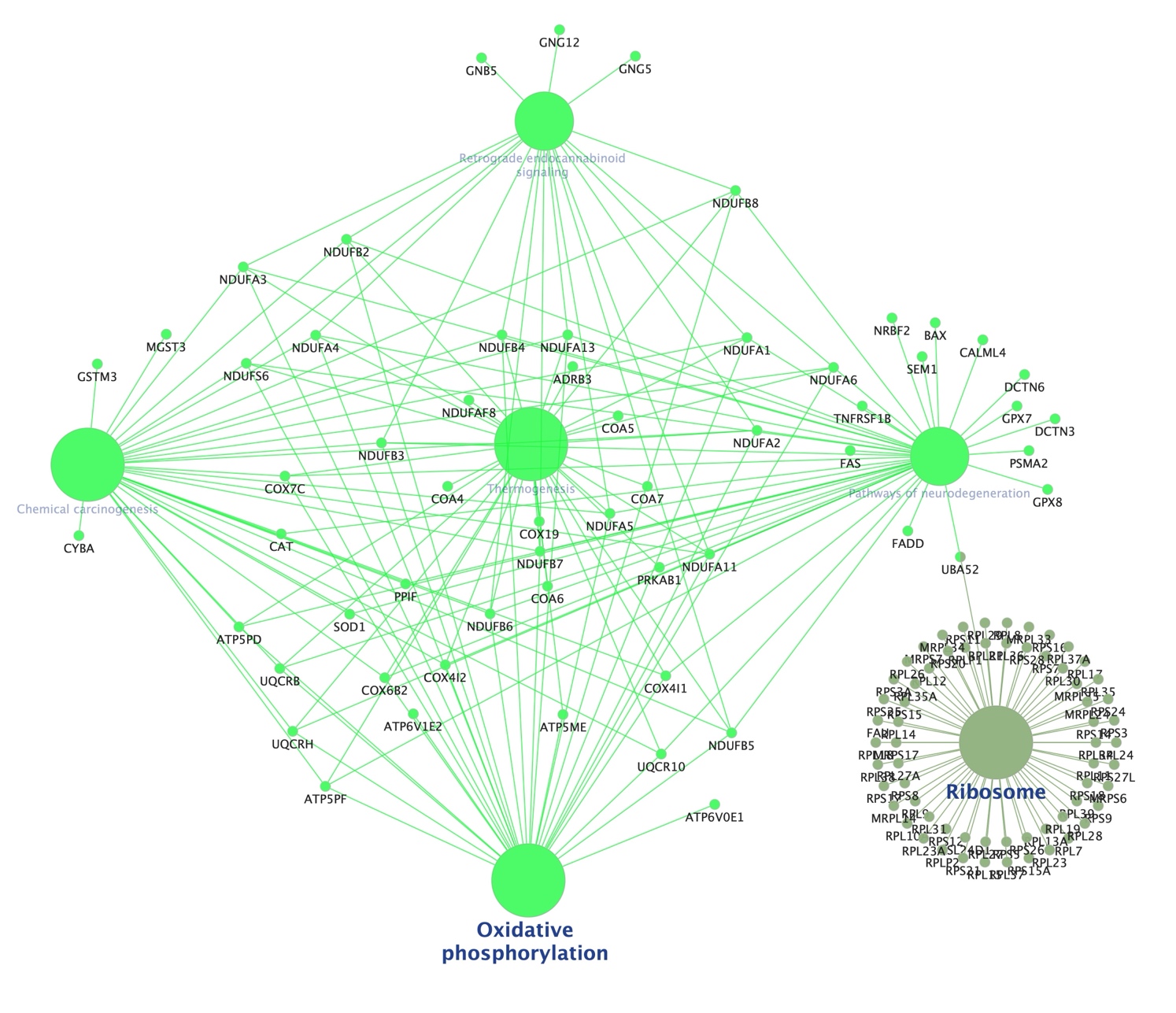


**Figure S4.** Network visualization of the enriched KEGG pathways and constituent genes downregulated in pituitaries of ethynylestradiol (250 nmol/kg bw) treated female Atlantic cod. Pathway enrichment and network visualization was performed in Cytoscape using the GlueGo application.


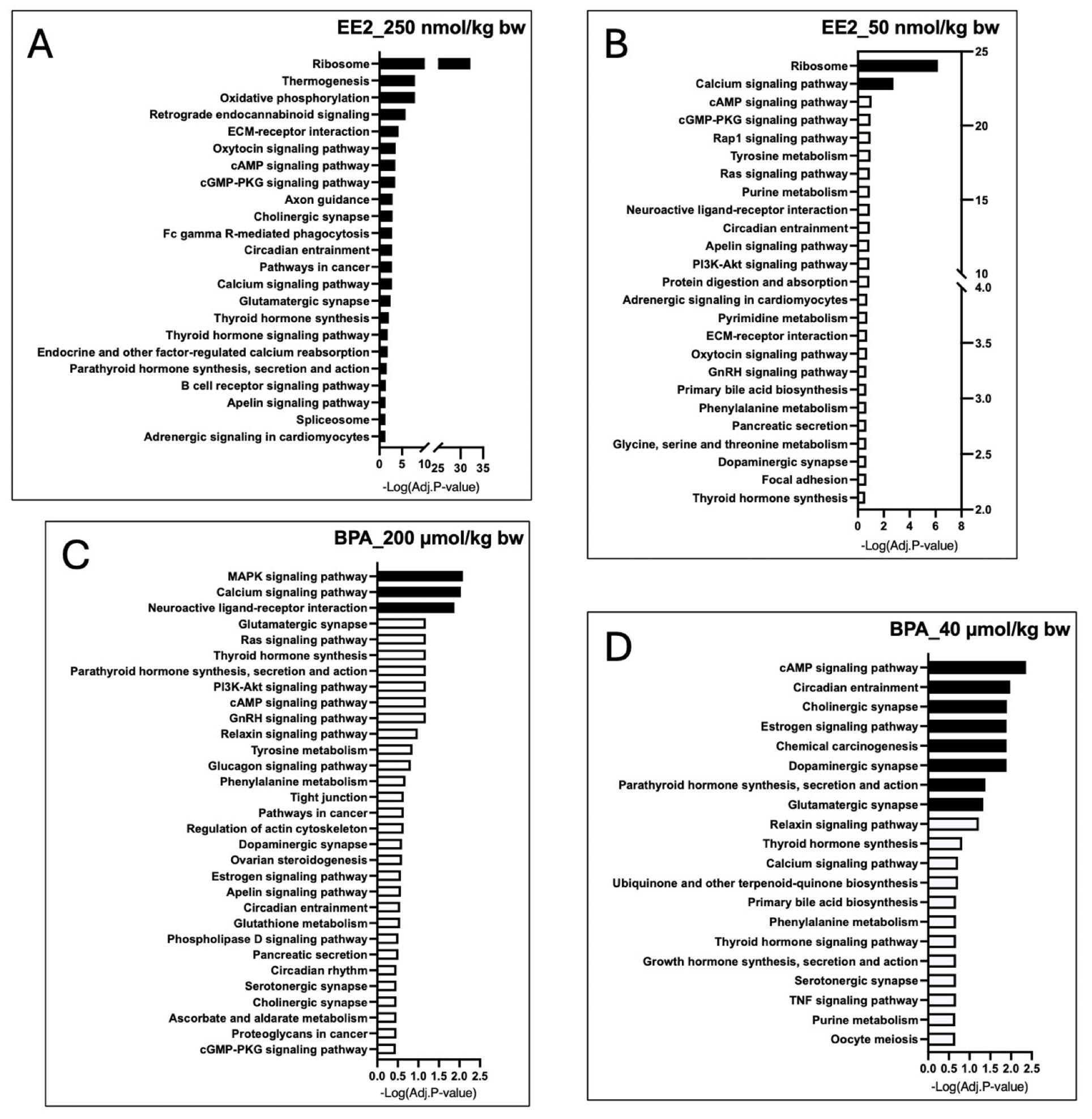


**Figure S5.** Enriched KEGG pathways in pituitary DEGs from medium and high doses of EE2 (**A** and **B**) and BPA (**C** and **D**). Filled bars indicate pathways with significant enrichment (adjusted p-value < 0.05).


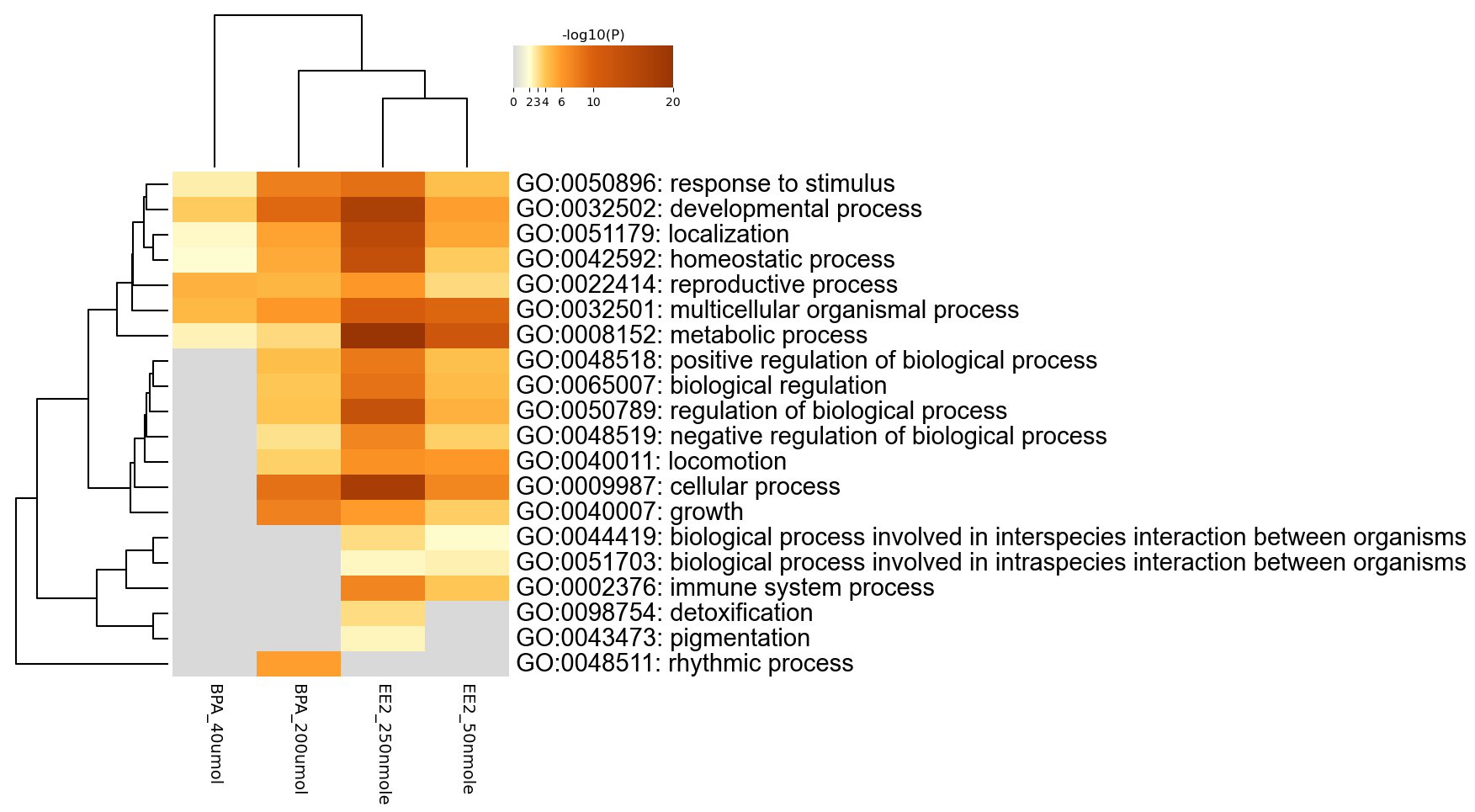


**Figure S6.** Heatmap comparison of top-level Gene Ontology (GO) biological processes enriched in differentially enriched genes in pituitary of juvenile female Atlantic cod treated with BPA (40 and 200 μmol/kg bw) and EE2 (50 and 250 nmol/kg bw). The color scale (top right) indicates significance levels (negative log10 p-values) of the enriched pathways and processes. Enrichment analysis was performed in Metascape. BPA_40umol: bisphenol A, 40 μmol/kg bw; BPA_200umol: bisphenol A, 200 μmol/kg bw; EE2_50nmol: ethynylestradiol, 50 nmol/kg bw; EE2_250nmol: ethynylestradiol, 250 nmol/kg bw: body weight.

**
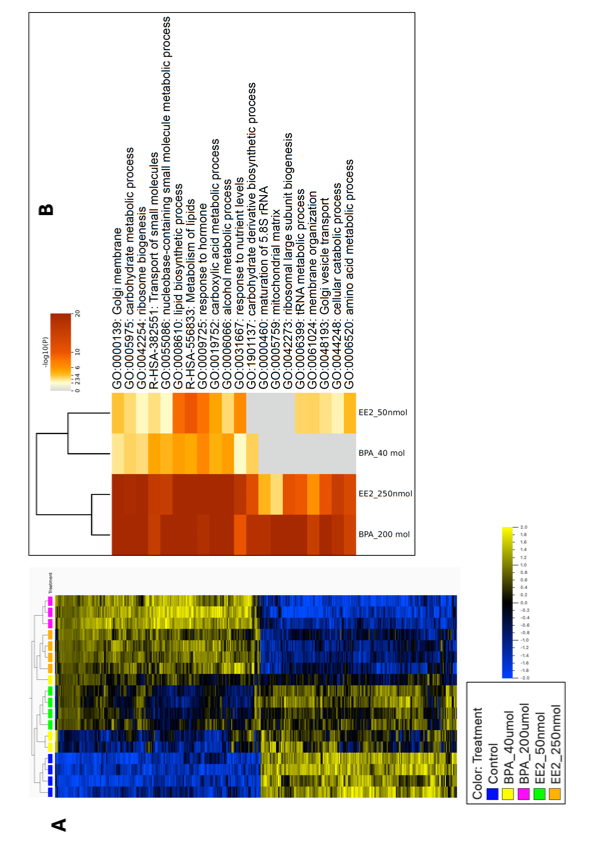
**

**Figure S7.** Heatmap comparison of top differntally expressed genes and enriched pathways in the liver of Atlantic cod treated with estrogenic BPA and EE2 for 72 h. (**A**) Hierarchical cluster analysis the top differentially expressed genes (Qlucore, ANOVA, q-value < 0.01) in different treated groups shown (bottom lower left). The yellow and blue ends of the color bar (bottom) indicate highest and lowest relative expression levels, respectively. (**B**) Heatmap comparison of top 20 enriched pathways and processes in the treated groups. The indicated doses of BPA and EE2 shown on top are all per kg bw. The color scale (top right) indicates significance levels (negative log10 p-values) of the enriched pathways and processes.


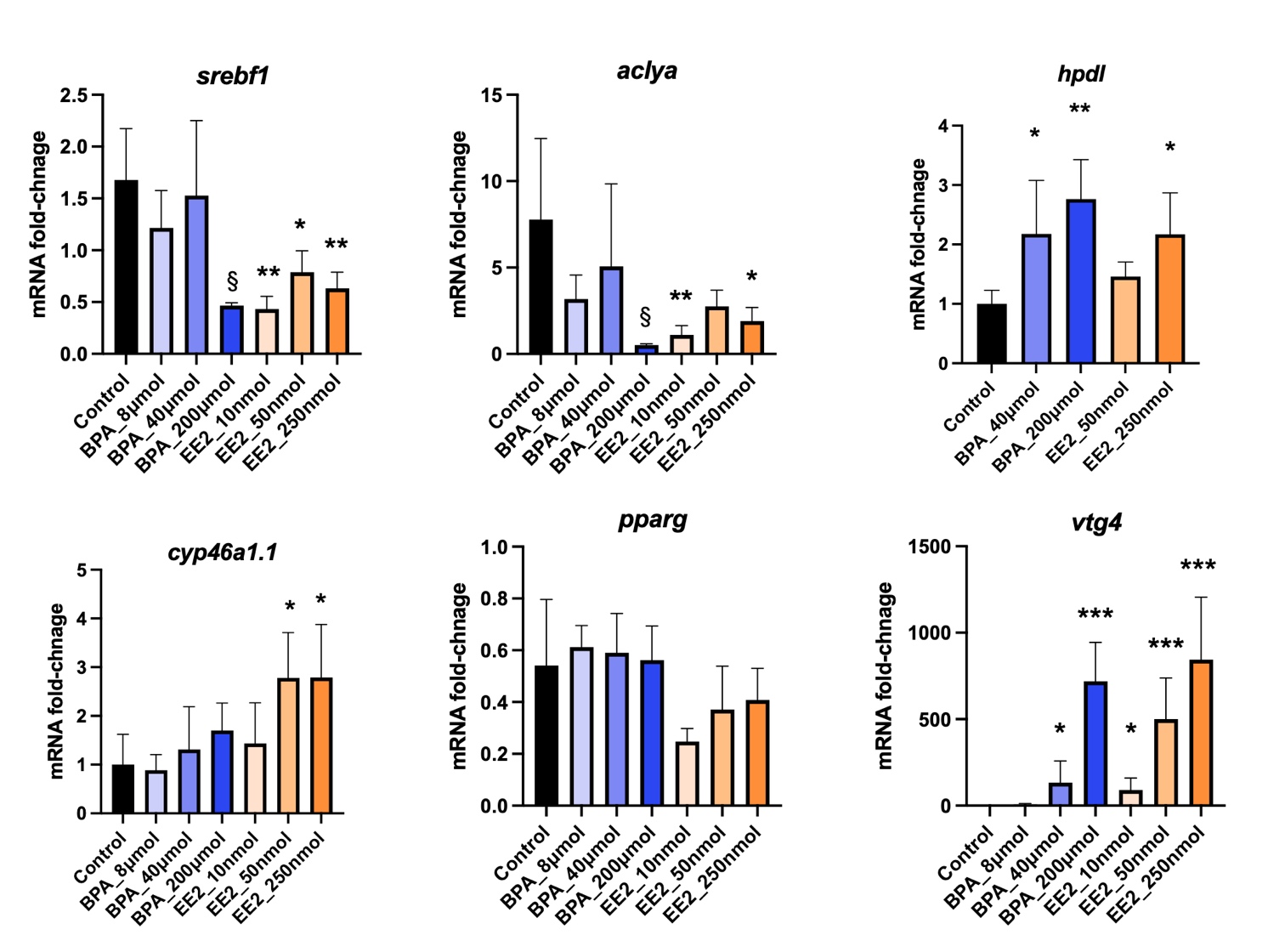


**A**

**B**

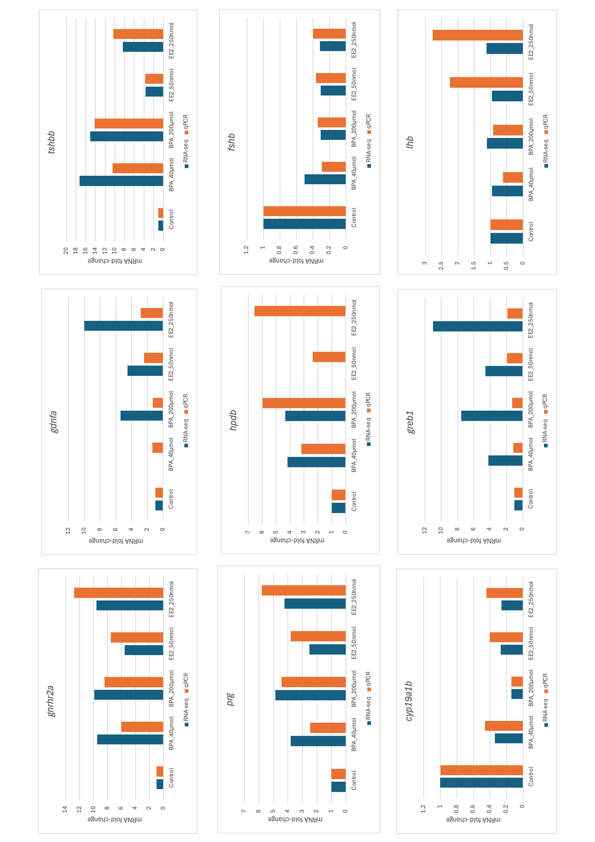


**C**


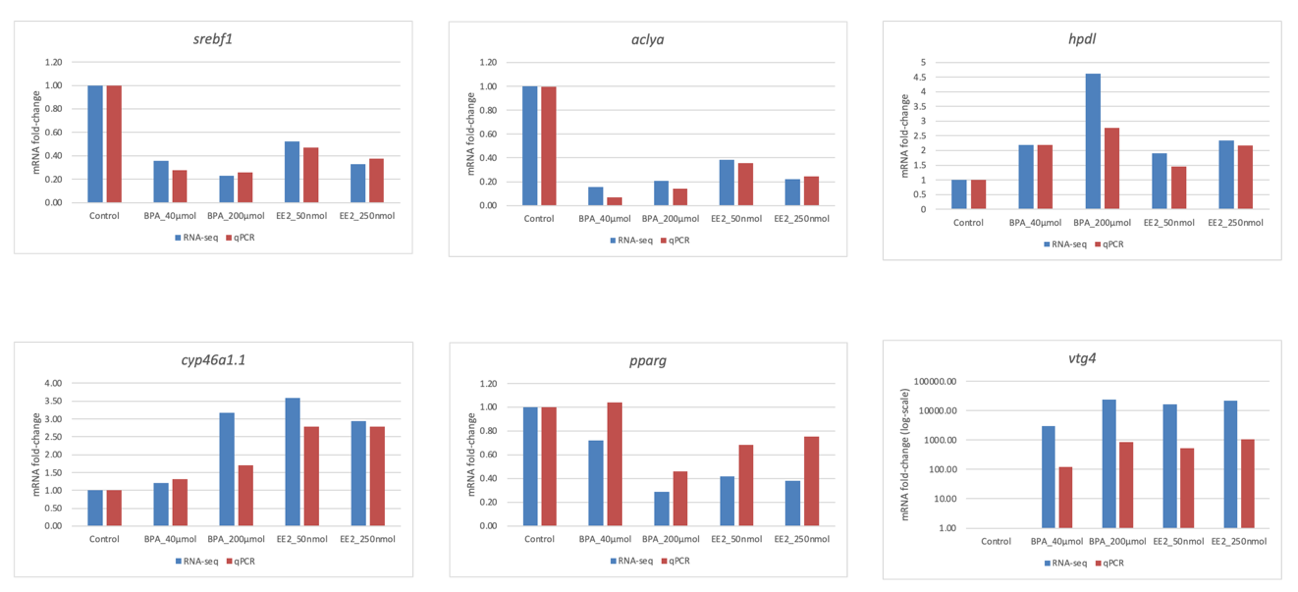


**D**

**Figure S8.** qPCR assay of selected genes differentially expressed in the liver of Atlantic cod treated with indicated doses (per kg bw) of BPA and EE2 (**A**).  Plasma Vitellogenin (Vtg) levels determined using ELISA (**B**). Comparison of expression fold-changes of expression for each gene obtained in RNA-seq and qPCR assays in the pituitary (**C**) and the liver (**D**). *n = 3-6* for all groups except the 200 μmol BPA group for *srebf1* and *aclya* genes in **A, B** and **C** (indicated by §, where n=2). *n = 3-6* for all groups in **D**. Data represent mean ± sd. Asterisk (*) indicates statistically significant difference from the control group (**A** and **B**. **p < 0.05, **p < 0.01, ***p < 0.001*.


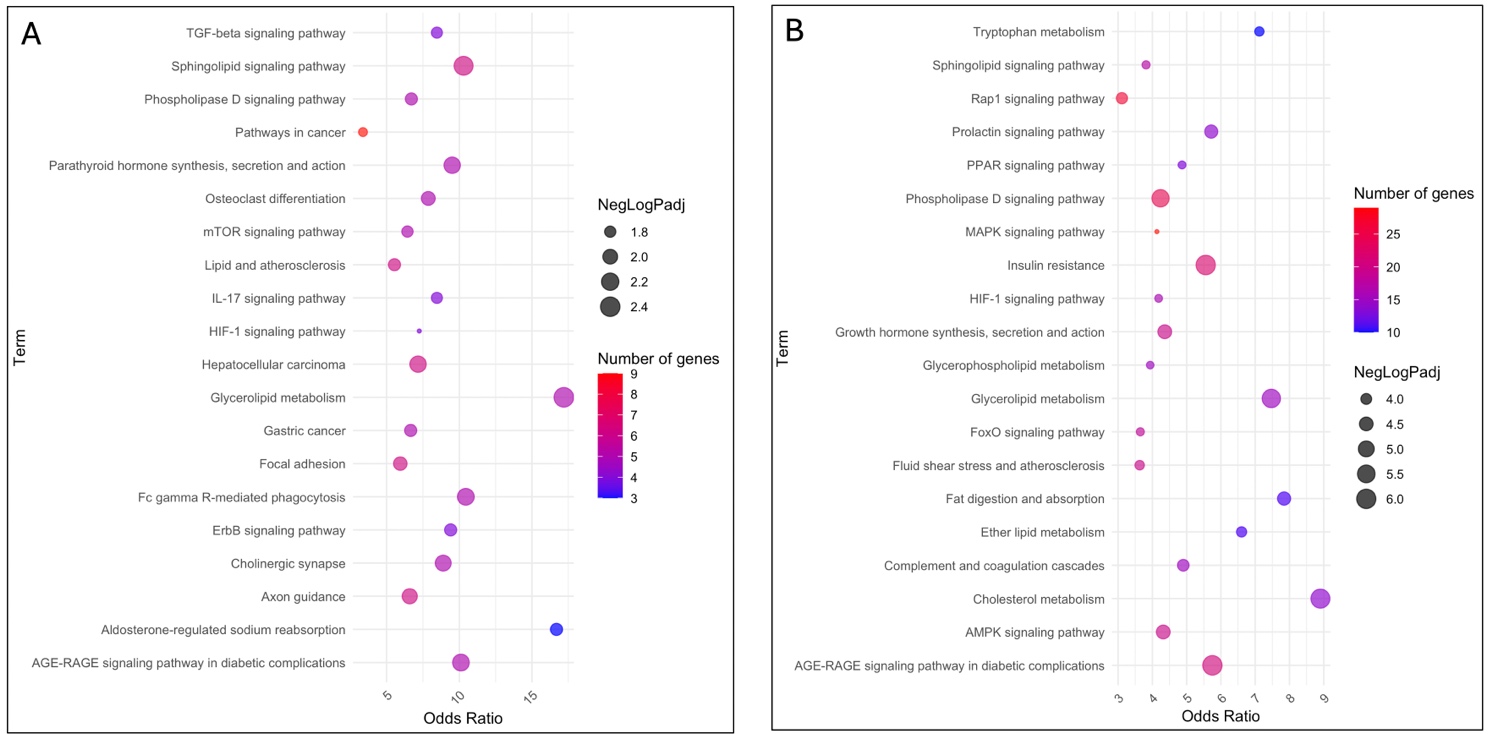


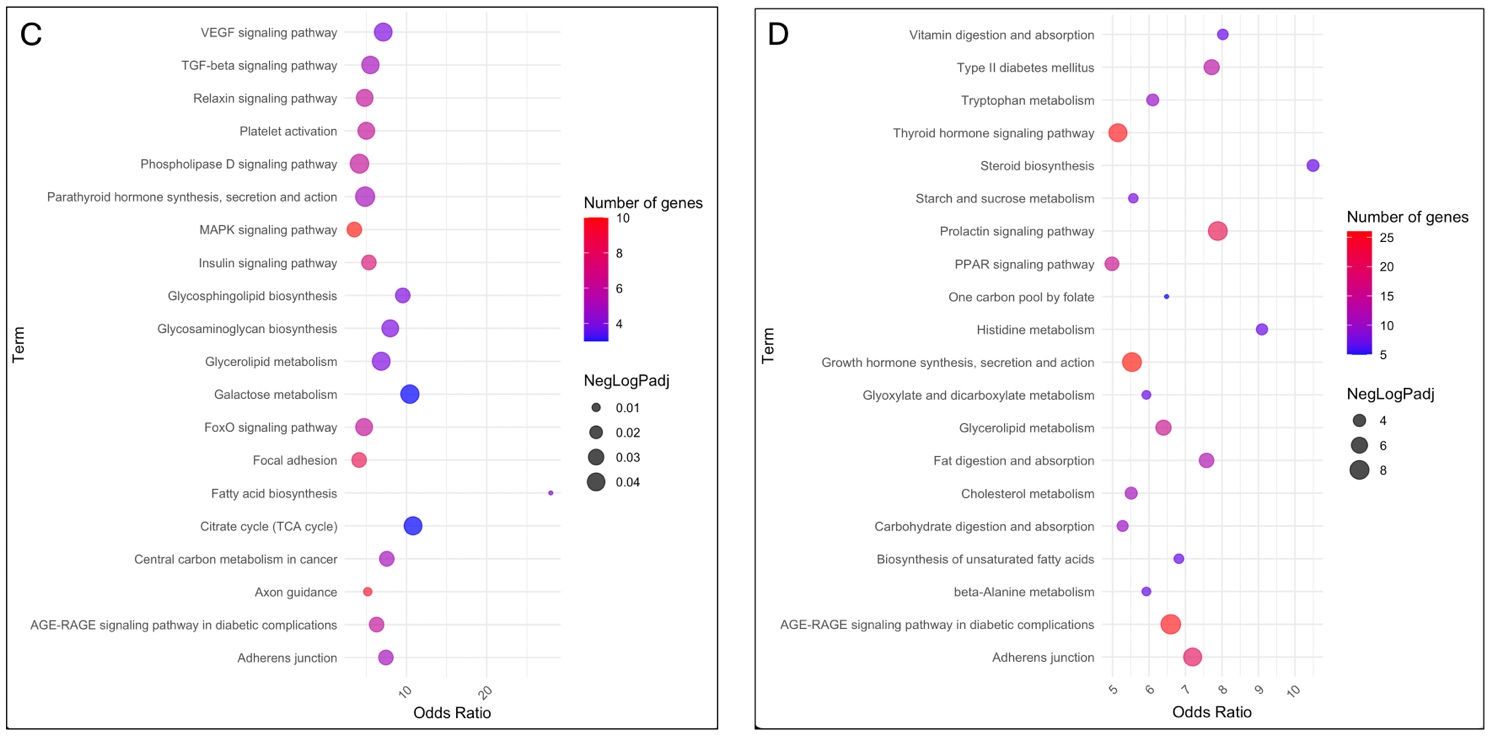


**Figure S9.** Top 20 enriched KEGG pathways in downregulated genes from medium and high doses of EE2 (**A** and **B**) and BPA (**C** and **D),** respectively. Downregulated genes in the list of DEGs in the liver of Atlantic cod treated with ethynylestradiol at medium (50 nmol/kg bw) (**A**) and high (250 nmol/kg bw) (**B**), bisphenol A at medium (40 μmol/kg bw) (**C**) and high (200 μmol/kg bw) (**D**) were were analyzed in Enrichr and the top 20 significantly enriched (*p* < 0.05) KEGG pathways were plotted. NegLogPadj: negative logarithm of adjusted p-values.


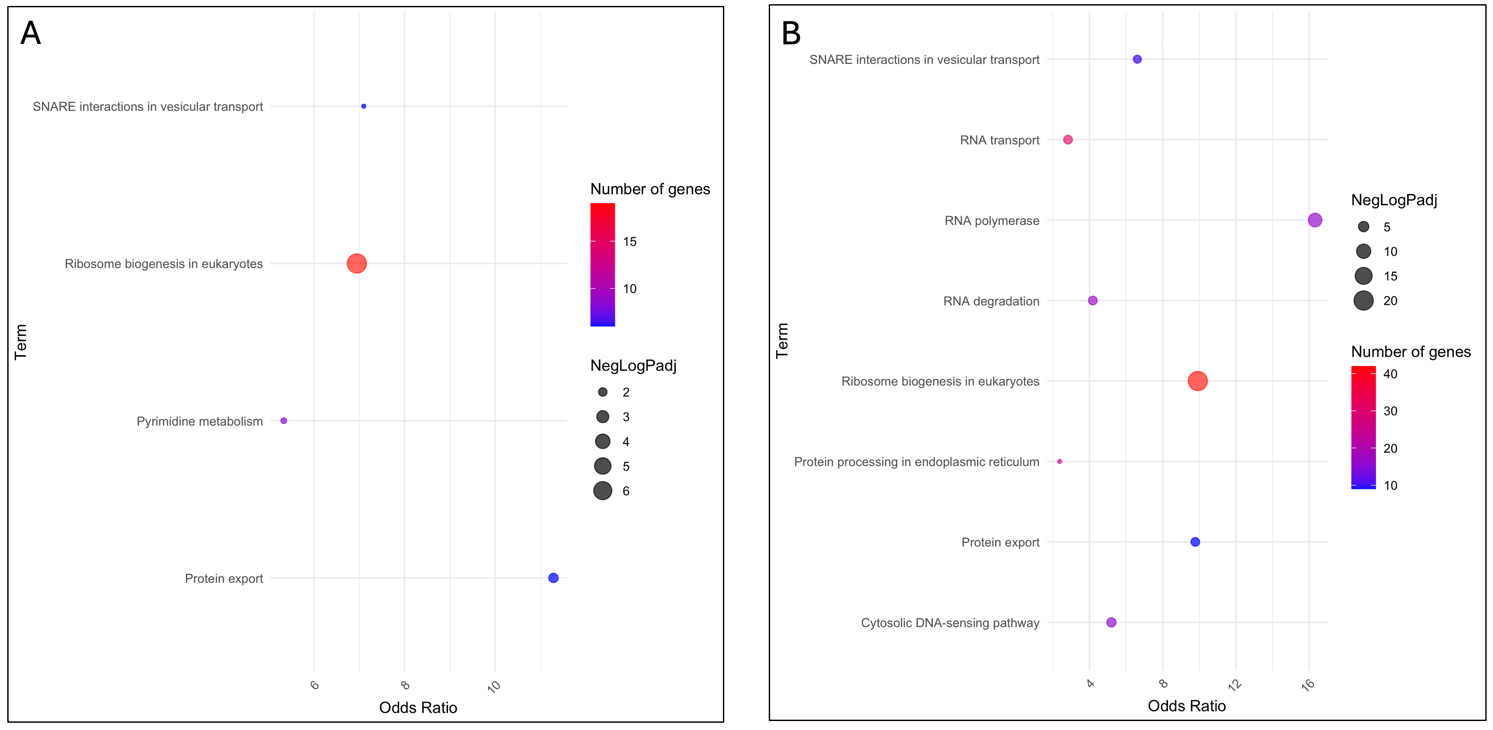


**Figure S10.** Significantly enriched KEGG pathways in upregulated genes from high dose of EE2 (**A)** and BPA ( **B**). Upregulated genes in the list of DEGs in the liver of Atlantic cod treated with ethynylestradiol at high (250 nmol/kg bw) (**A**) and bisphenol A at high (200 μmol/kg bw) (**B**) were were analyzed in Enrichr and the significantly enriched (*p* < 0.05) KEGG pathways were plotted. NegLogPadj: negative logarithm of adjusted p-values.


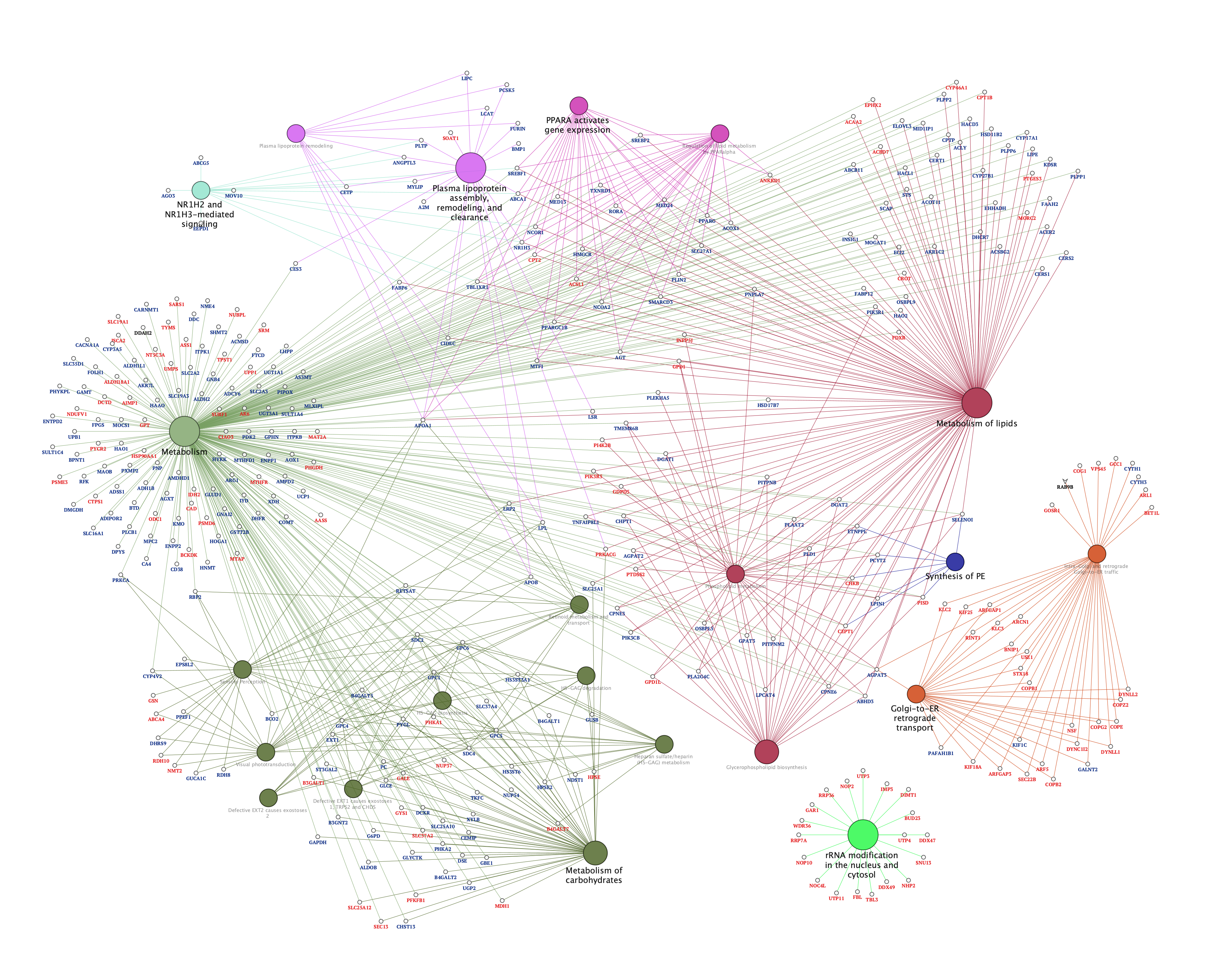


**Figure S11.** Network visualization of enriched pathways and populating genes differentially expressed in the liver of Atlantic cod treated with ethynylestradiol (250 nmol/kg body weight). Significantly (*p* < 0.05) enriched networks and KEGG, Wikipathways and Reactome pathways generated in Cytoscape using the ClueGo application are shown. The size of a pathway node is proportional to the number populating genes. The pathway term nodes belonging to similar pathways share same color, and the most important term is in bold face. Th symbols of upregulated and down-regulated genes are colored red and blue, respectively.

**
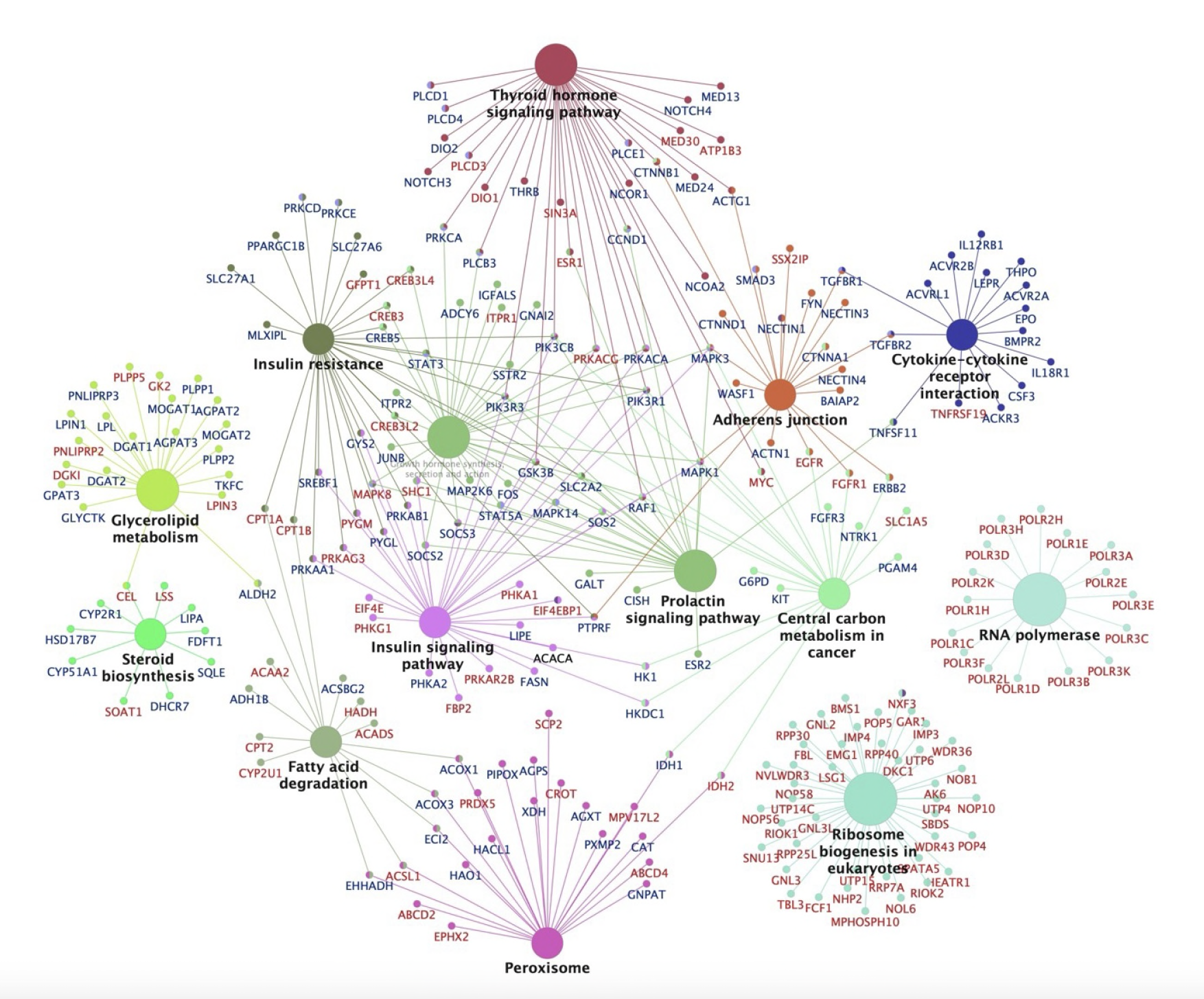
**

**Figure S12.** Network visualization of enriched pathways and populating genes differentially expressed in the liver of Atlantic cod treated with bisphenol A (200 μmol/kg bwt). Significantly enriched (adjusted *p-value* < 0.05) networks and KEGG pathways generated in Cytoscape using the ClueGo application are shown. The size of a pathway node is proportional to the number populating genes. The pathway term nodes belonging to similar pathways share same color, and the most important term is in bold face. The symbols of upregulated and down-regulated genes are colored red and blue, respectively.


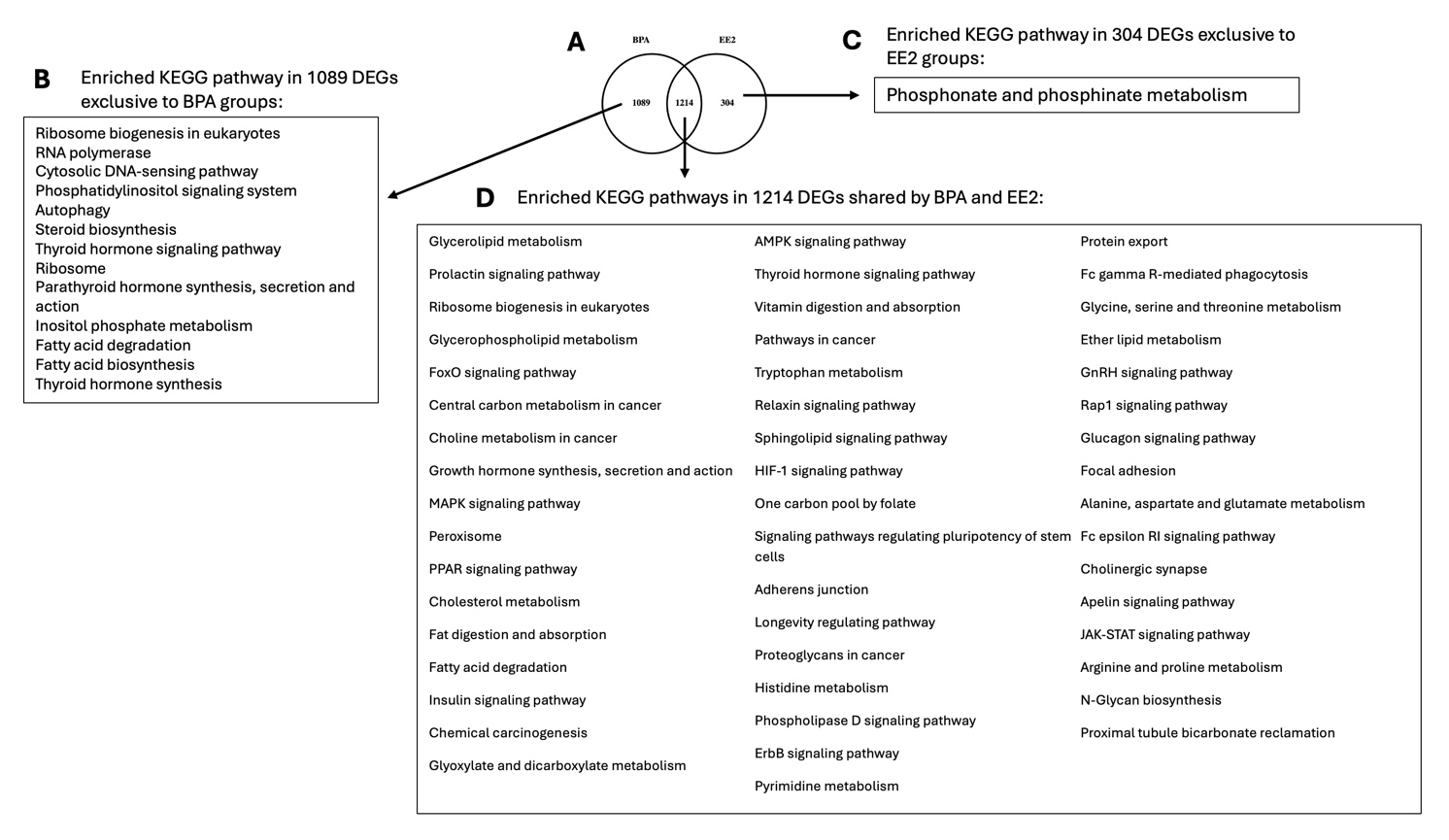


**Figure S13.** Venn diagram comparison of the lists of liver DEGs (with human orthologs) in BPA and EE2 groups (**A**). KEGG pathways enriched in 1089 genes exclusive to BPA (**B),**  KEGG pathway enriched in 304 genes exclusive to EE2 (**C**) and KEGG pathways enriched in 1214 shared genes (**D)**. Note that DEGs without human orthologs are not included in the comparisons. Enrichment analysis was performed in Enrichr (https://maayanlab.cloud/Enrichr/).
